# Discovering hematoma-stimulated circuits for secondary brain injury after intraventricular hemorrhage by spatial transcriptome analysis

**DOI:** 10.3389/fimmu.2023.1123652

**Published:** 2023-02-07

**Authors:** Le Zhang, Jiayidaer Badai, Guan Wang, Xufang Ru, Wenkai Song, Yujie You, Jiaojiao He, Suna Huang, Hua Feng, Runsheng Chen, Yi Zhao, Yujie Chen

**Affiliations:** ^1^ College of Computer Science, Sichuan University, Chengdu, China; ^2^ Innovation Center of Nursing Research, West China Hospital, Sichuan University, Chengdu, China; ^3^ Chinese Academy of Sciences (CAS) Key Laboratory of Separation Science for Analytical Chemistry, Dalian Institute of Chemical Physics, Chinese Academy of Sciences, Dalian, China; ^4^ Department of Neurosurgery and State Key Laboratory of Trauma, Burn and Combined Injury, Southwest Hospital, Army Medical University, Chongqing, China; ^5^ Center for Big Data Research in Health, Institute of Biophysics, Chinese Academy of Sciences, Beijing, China; ^6^ West China Biomedical Big Data Center, West China Hospital, Sichuan University, Chengdu, China; ^7^ Research Center for Ubiquitous Computing Systems, Institute of Computing Technology, Chinese Academy of Sciences, Beijing, China

**Keywords:** intraventricular haemorrhage, neural circuits, spatial transcriptome sequencing, bioinformatics analysis, secondary brain injury

## Abstract

**Introduction:**

Central nervous system (CNS) diseases, such as neurodegenerative disorders and brain diseases caused by acute injuries, are important, yet challenging to study due to disease lesion locations and other complexities.

**Methods:**

Utilizing the powerful method of spatial transcriptome analysis together with novel algorithms we developed for the study, we report here for the first time a 3D trajectory map of gene expression changes in the brain following acute neural injury using a mouse model of intraventricular hemorrhage (IVH). IVH is a common and representative complication after various acute brain injuries with severe mortality and mobility implications.

**Results:**

Our data identified three main 3D global pseudospace-time trajectory bundles that represent the main neural circuits from the lateral ventricle to the hippocampus and primary cortex affected by experimental IVH stimulation. Further analysis indicated a rapid response in the primary cortex, as well as a direct and integrated effect on the hippocampus after IVH stimulation.

**Discussion:**

These results are informative for understanding the pathophysiological changes, including the spatial and temporal patterns of gene expression changes, in IVH patients after acute brain injury, strategizing more effective clinical management regimens, and developing novel bioinformatics strategies for the study of other CNS diseases. The algorithm strategies used in this study are searchable via a web service (www.combio-lezhang.online/3dstivh/home).

## 1 Introduction

Intraventricular hemorrhage (IVH) refers to bleeding inside the ventricles of the brain or a hematoma dislodging from the periventricular brain parenchyma, which is usually secondary to traumatic brain injury, subarachnoid hemorrhage, or spontaneous intracerebral hemorrhage. IVH is one of the common complications after various acute brain injuries (1–3) that causes severe mortality and mobility dysfunction and is a great economic and societal burden. To date, the gold standard for the treatment of IVH is hematoma removal and hydrocephalus prevention (4), which relieves the general pathophysiological effects and elevated intracranial pressure in the lateral walls of the ventricles (3) as well as reduces the obstructions and inflammatory responses from blood metabolic stimulations (5, 6). However, a recently completed large-scale randomized clinical trial (CLEAR III, Trial No. NCT00784134) employing alteplase to effectively clear intraventricular hematomas did not demonstrate adequate neurobehavioral benefits for IVH patients (7–9). With the development of neurosurgery and the improvement of neurocritical care, the direct IVH mortality rate has gradually decreased. However, we still need to understand the neurological dysfunction that occurs after IVH in order to alleviate it and produce better outcomes in these patients. Indeed, the pathophysiology of how these hematoma stimulations in the ventricles cause neurological dysfunction remains unknown.

For this reason, this study investigated the pathophysiological mechanism of secondary neurological dysfunction after IVH and the related intervention strategies by developing a bioinformatics analysis workflow based on spatial transcriptome sequencing (10, 11). First, by constructing 3D pseudospace-time trajectories (12, 13), we identified the cell subtypes (14) generated after IVH stimulation and their potentially similar cell types. We then explored and validated the important pathophysiological mechanisms for these cell subtypes by carrying out cell–cell communication strength (12, 13) and pathway analyses (15–19).

In summary, our study not only identified the brain regions affected by secondary neurological dysfunction after IVH stimulation from a 3D perspective but also allows for the further investigation of driving cell types and molecular pathways, which might benefit the construction of translational intervention strategies for secondary brain injuries after IVH at the molecular and cellular levels in the distant future (20–22). Finally, our study is available *via* a web service (www.combio-lezhang.online/3dstivh/home).

## 2 Materials and methods

### 2.1 Experimental setup

Five wild-type C57/BL6 male mice (weighing 25 ± 5 g, 8 weeks old, from the Experimental Animal Center of the Third Military Medical University, Chongqing, China) were used in this study. These mice were housed in a temperature-controlled room under specific-pathogen-free conditions and a standard 12-h light/dark cycle, with *ad libitum* access to food and water. All experiments were reported in compliance with the Animal Research: Reporting *In Vivo* Experiments (ARRIVE) guidelines. The experimental protocols were approved by the Laboratory Animal Welfare and Ethics Committee of the Third Military Medical University (AMUWEC2020762) and performed according to the Guide for the Care and Use of Laboratory Animals.

An IVH model was established according to previously described methods with modified coordinates. Briefly, mice in each group were deeply anesthetized by an intraperitoneal injection of 2 mg of ketamine and 0.4 mg of xylazine in 0.9% saline. A feedback-controlled heating pad was used to maintain body temperature at 37.0°C. A small cranial burr hole was drilled, and a 32-gauge needle was inserted stereotaxically into the right lateral ventricle (coordinates: 1.0 mm lateral, 0.24 mm posterior, and 2.5 mm ventral to the bregma) to establish the IVH model under stereotactic guidance. Approximately 25 μl of autologous blood was then injected at a rate of 5 μl/min using a microinfusion pump (Harvard Apparatus, Holliston, MA, USA). The burr hole was sealed with bone wax, and the skin incision was closed with sutures after the needle was removed. Sham-operated mice underwent the same surgery without blood injection. The brains of the mice were harvested at different time points after IVH stimulation, after they were deeply anesthetized for further study.

### 2.2 Visium spatial transcriptome sequencing

We collected wild-type C57/BL6 mouse brains, which were classified into the no-surgery group (control), the needle-puncture group without blood injection (sham), and the needle-puncture group with blood injection on the first, third, and seventh days. These brains were then frozen on dry ice in an optimal cutting temperature (OCT) compound.

Frozen brains were then sectioned coronally at 10 μm on a cryostat and mounted on 10X Genomics Visium spatial transcriptome slides. The tissue locations of the library patches started from the emergence of the lateral ventricle, with 20 μm intervals and a 10-μm margin of error (**SM Table 1**).

For morphological analysis and spatial alignment of sequencing data, sections were fixed in methanol at −20°C for 30 min before hematoxylin and eosin (H&E) staining. Additionally, all fractions were measured using a bioanalyzer (Agilent 2100) meeting the RNA integrity number RIN>=7.

After brightfield imaging, we permeabilized 19 brain sections, the permeabilization time of which are listed in **SM Table 1**. After tissue permeabilization, we isolated poly-A mRNA at each point in the capture region and added spatial barcodes and unique molecular identities into reads for library construction.

A DNF-915 kit and our library detection instrument were employed for quantification by qPCR. After the library was quantified, we performed PE150 paired-end sequencing using the Illumina NovaSeq 6000 S4 sequencing platform with 50,000–100,000 reads per locus (spot).

Finally, genomes and images were aligned to the mouse reference genome mm10-3.0.0 by SpaceRanger (v1.1.0) software. The data were analyzed and normalized by using Seurat (v3.1.2) software and the transform package, respectively.

### 2.3 Cell subtype analysis

#### 2.3.1 Cell subtype identification

We used the top 30 up/downregulated 3D transition genes for each 3D subtrajectory (**SM Table 1** and **SR3 Table 2**) as input. We then employed our developed algorithm (**SM Tables 4, 5**) to identify a cell subtype that corresponded to the selected trajectory at different times (**SM Tables 4, 5** and **SR4 Table 1**).

#### 2.3.2 Similarity algorithm for cell subtypes

We used the marker gene sets of cell subtypes corresponding to the selected trajectory at different times (**SM Tables 4, 5** and **SR4 Table 2**) as input. We then employed a similarity algorithm for cell subtypes (**SM Tables 4, 6**) to describe the similarity between cell subtypes at different times within the same trajectory (**SM Tables 4, 6** and **SR4 Table 3**).

#### 2.3.3 Search for similar cell types by CellMeSH

Here, we used marker genes of the cell subtypes as input. We then found the top 5 similar cell types for each cell subtype by using the CellMeSH database (23). Next, we performed a union operation for these top 5 similar cell types.

### 2.4 Cell−cell communication analysis

We use $Z{\text{score}}_{i}^{{\text{LR}}_{n}}$ (**SM Tables 4, 6** and **SR5 Table 2**) for each ligand−receptor pair (LR*_n_*) of cell subtypes and $Z{\text{score}}_{i}^{{\text{LR}}_{n}}$ for all the brain regions as the input. We then employed the cell−cell communication strength (density) algorithm (**SM Tables 7, 8**) to obtain the discrete interaction intensity ( $ZT_{i}^{{\text{LR}}_{n}}$ ) after conversion of the continuous interaction intensity $\left(Z{\text{score}}_{i}^{{\text{LR}}_{n}}\right)$ and the sum of the interaction intensity $\left(ZT_{i}^{{\text{LR}}_{n}}\right)$ of *N* points in the corresponding position (*ZT*Total) (**SM Tables 7, 8** and **SR5 Table 3**) to obtain the average Density^LR^ *^n^* for each ligand–receptor pair of the cell subtypes (**SM Tables 7, 8** and **SR5 Table 4**).

### 2.5 Signaling pathway analysis

We used mutual pathway sets for our identified cell subtypes ( *P*_*A*_Trajectory_*n*__, *n* = 1 … *N* ) (**SM Tables 7, 8** and **SR6 Table 2**) and similar cell types ( *P*_*B*_Trajectory_*n*__, *n* = 1 … *N* ) (**SM Tables 7, 8** and **SR6 Table 3**) as the input. We then employed the similarity algorithm for mutual pathway sets (**SM Tables 9, 10**) to obtain the mutual pathway set ( *P*_*inter*_Trajectory_*n*__ ) (**SM Tables 9, 10** and **SR6 Table 4**) and the similarity ( ${\text{Similarity}}_{{\text{Trajectory}}_{n}}^{P_{A\_B}}$ ) between *P*_*A*_Trajectory_*n*__ and *P*_*B*_Trajectory_*n*__ (*n* = 1 … *N*).

## 3 Results

### 3.1 Developing a spatial transcriptome sequencing-based bioinformatics analysis workflow to investigate IVH

**Figure 1:**
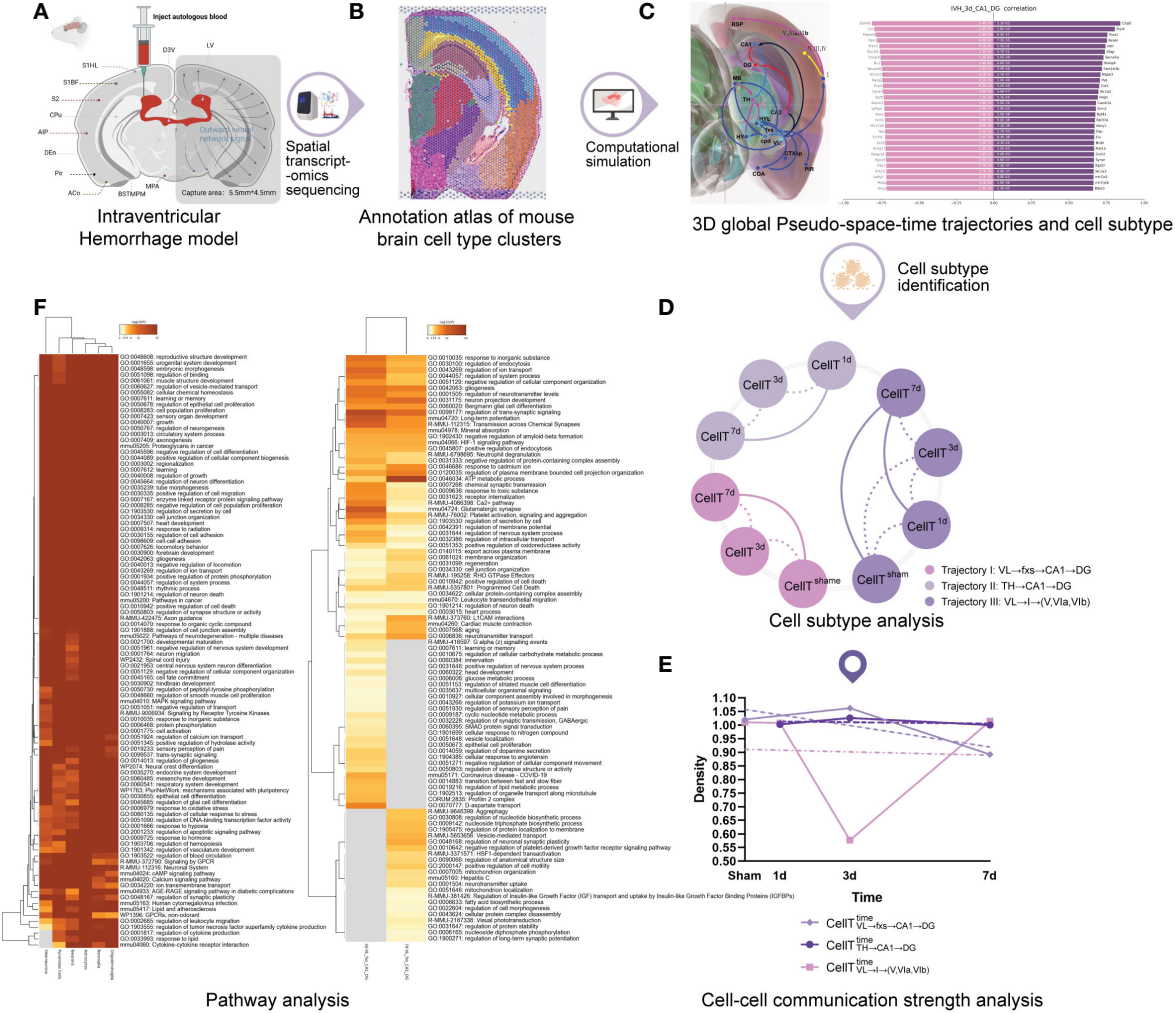
The workflow of the study. **(A)** Secondary intraventricular hemorrhage (IVH) experiment. **(B)** Annotation atlas of mouse brain cell-type clusters. **(C)** 3D global pseudospace-time trajectories and the transition gene sets. **(D)** Cell subtype analysis. **(E)** Cell−cell communication strength analysis. **(F)** Pathway analysis.

To comprehensively dissect the complex gene expression changes following acute brain injury, we first developed a spatial transcriptomics sequencing-based bioinformatics analysis workflow to investigate IVH (**Figure 1**).

First, given the difficulty of conducting the analysis with human patient samples, we constructed an IVH model (**Figure 1A**) in mice by injecting autologous blood into the lateral ventricle (for technical details, please refer to Experimental procedures).

Spatial transcriptomics sequencing (10, 11) and the spatial and morphological expression (SME) clustering algorithm (12) were subsequently used to analyze frozen sections of IVH model mouse brain to obtain the annotation atlas of cell type clusters (**Figure 1B**; Visium spatial transcriptome sequencing of the Methods section) and to construct 3D global pseudospace-time trajectories (**Figure 1C**) and transition gene sets. More extensive cell subtype characterizations were performed on the three identified 3D global pseudospace-time trajectories at different times (**Figure 1D**). We further explored the molecular-level changes in these cell subtypes (**Figure 1E**) by using a newly developed cell–cell interaction intensity and density algorithm and evaluated the biological cha++racteristics of these cell subtypes using pathway analysis (**Figure 1F**).

### 3.2 3D global pseudospace-time trajectory reconstruction for mouse brain tissue after lateral ventricle hemorrhage

**Figure 2:**
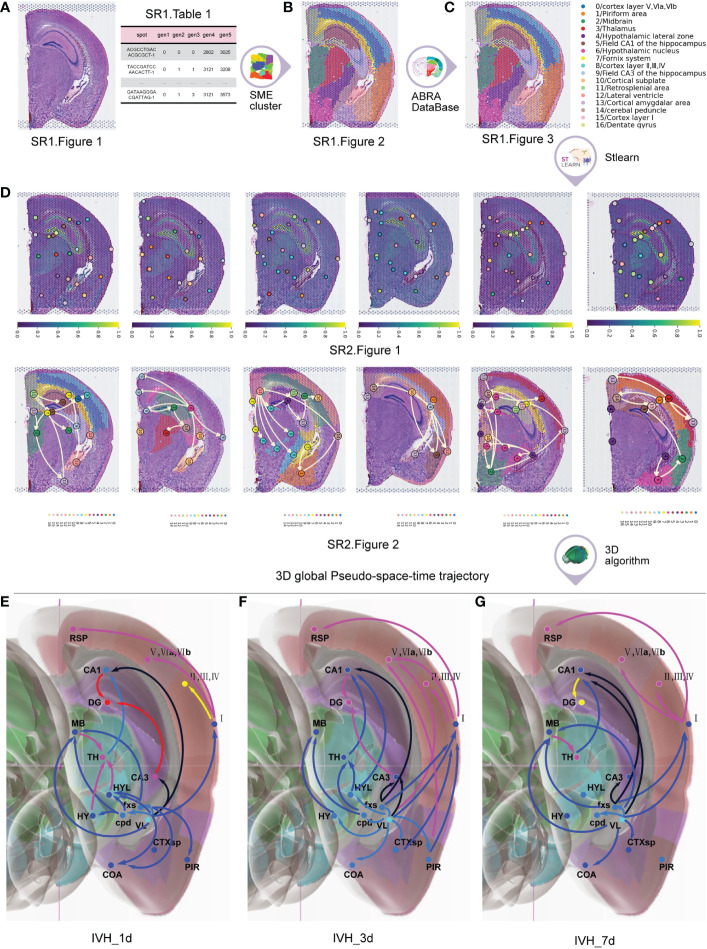
The process to obtain the 3D global pseudospace-time trajectory after IVH. **(A)** Hematoxylin and eosin (H&E)-stained sections and spatial gene expression. **(B)** Normalization and clustering of the cell types with similar gene expression. **(C)** Annotation atlas of cell-type clusters. **(D)** Planar diffusion pseudotime plots and planar global pseudospace-time trajectories. **(E)** The 3D global pseudospace-time trajectories on the first day after IVH stimulation. **(F)** The 3D global pseudospace-time trajectories on the third day after IVH stimulation. **(G)** The 3D global pseudospace-time trajectories on the seventh day after IVH stimulation.

Initially, we used 19 original frozen sections from the brains of five mice with IVH stimulation as the input. After carrying out spatial transcriptome sequencing (10, 11), we performed H&E staining on the sections and examined spatial gene expression (**Figure 2A** (**SR1 Figure 1**; **SR1 Table 1**).

Next, we employed the SME algorithm (12) to normalize and cluster the cell types with similar gene expressions (**Figure 2B** (**SR1 Figure 2**)) by inputting (**SR1 Figure 1** and **SR1 Table 1**).

**Figure 3:**
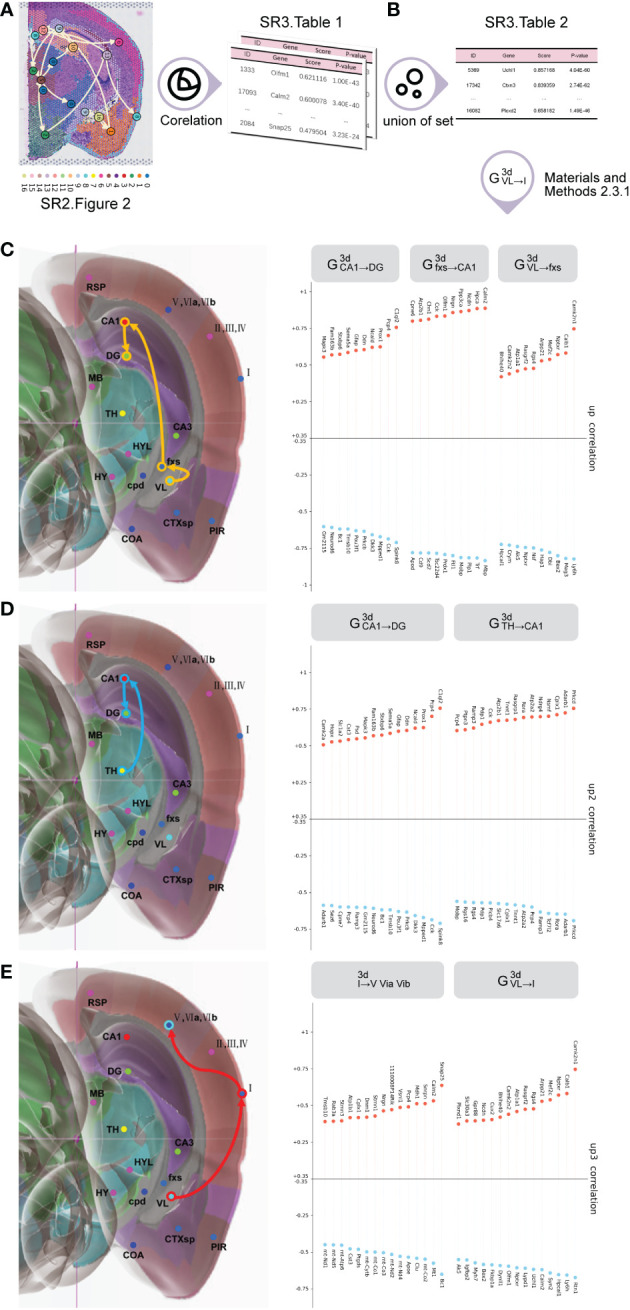
The process to obtain trajectory-based transition gene sets for 3D global pseudospace-time trajectories. **(A)** The top 30 planar up/downregulated transition genes for each planar subtrajectory. **(B)** The top 30 3D up/downregulated transition genes for each 3D subtrajectory. **(C)** The top 30 3D trajectory-based transition gene sets of VL→fxs→CA1→DG. **(D)** The top 30 3D trajectory-based transition gene sets of TH→CA1→DG. **(E)** The top 30 3D trajectory-based transition gene sets of VL→I→(V, VIa, VIb).

Following that, we obtained the annotation atlas of cell type clusters (24) (**Figure 2C**) in different brain regions by comparing the clustering results with the corresponding anatomical brain regions in the Allen Brain Reference Atlas map (25), which describes the whole transcriptional signal in the brains of mice after IVH stimulation (**SR1 Figure 3**).

We then employed the stLearn algorithm (12) to obtain the planar diffusion pseudotime plots and the planar global pseudospace-time trajectories (**Figure 2D** (**SR2 Figures 1**, **2**)) by inputting (**SR1 Figure 3**).

Finally, we used a 3D global pseudospace-time trajectory reconstruction algorithm that we developed (**SM Tables 2, 3**) to obtain 3D global pseudospace-time trajectories (**Figures 2E–G**) by inputting **Figures 2D** (**SR2 Figures 1**, **2**).

In addition to a disturbance in consciousness, it has been commonly observed that short-term motor dysfunction and long-term cognitive disorders occur after a single IVH. **Table 1** classifies the 3D global pseudospace-time trajectories (**Figures 2E–G**) into three major trajectory bundles based on their pivotal effect after hematoma stimulation and their representative neural circuits that are involved in motor dysfunction and cognitive disorder.

**Table 1:** Three major trajectory bundles from the lateral ventricle to the hippocampus and primary cortex after hematoma stimulation.

Trajectory bundles	Trajectories	Description
I	1. VL → CA1 → DG 2. VL → CA3 → DG 3. VL → fxs → CA1 → DG 4. VL → fxs → CA3 → DG 5. VL → fxs → CA3	Direct from the lateral ventricle to the hippocampus.
II	1. VL → COA 2. VL → fxs → COA 3. VL → MB → TH → CA1 → DG 4. VL → cpd → HY → TH → CA1 → DG 5. VL → cpd → MB → TH → CA1 → DG 6. VL → cpd → HYL → TH → CA1 → DG 7. VL → fxs → HYL → TH → CA1 → DG 8. VL → HYL → TH → CA1 → DG 9. VL → fxs → MB → TH → CA1 → DG 10. VL → HY → MB 11. VL → cpd → MB 12. VL → cpd → TH → CA1 → DG 13. VL → HYL → CA1 → DG 14. VL → fxs 15. VL → cpd → MB → TH → CA1 → DG	Dispersed signals from the lateral ventricle are integrated by the hypothalamus and sent to the hippocampus.
III	1. VL → I → (II, III, IV) 2. VL → I → (V, VIa, VIb) 3. VL → I → RSP 4. VL → fxs → PIR 5. VL → CTXsp → I → (II, III, IV) 6. VL → CTXsp → I → (V, VIa, VIb) 7. VL → CTXsp → I → RSP 8. VL → PIR → I → (II, III, IV) 9. VL → PIR → I → (V, VIa, VIb) 10. VL → PIR → I → RSP 11. VL → fxs → I →(II, III, IV) 12. VL → fxs → I →(V, VIa, VIb) 13. VL → fxs → I → RSP 14. VL → (V, VIa, VIb) 15. VL → PIR → (V, VIa, VIb) 16. VL →CTXsp	Direct from the lateral ventricle to the ipsilateral cortex.

Here, trajectory groups I and II are described in **Figures 2E–G** (**SR2 Figures S3.1A–C**, **3.2B, C**, **3.3B, C**) for different times, which extend from the lateral ventricle to the hippocampus. Trajectory bundle III is described in **Figures 2E–G** (**SR2 Figures 3.3A**, **3.3A, D**) for different times, which extend from the lateral ventricle to the primary cortex.

The following analysis of trajectory bundle III explores how hematoma stimulation in the lateral ventricle affects the cortex and causes motor dysfunction, while that of trajectory bundles I and II illustrates direct pathophysiological mechanisms of cognitive dysfunction after IVH, with unique neural circuits for hematoma stimulation in the lateral ventricle.

### 3.3 The trajectory-based transition gene set for 3D global pseudospace-time trajectories

Initially, we employed the Spearman’s correlation analysis to obtain the top 30 planar up/downregulated transition genes (**Figure 3A** (**SR3 Table 1**)) for each planar subtrajectory by inputting the planar global pseudospace-time trajectory (**SR2 Figure 2**) using stLearn software (12, 26–33).

Among the three major global pseudospace-time trajectory bundles (**Table 1**), we chose frequently appearing trajectories from each trajectory bundle at different times for further analysis, which are VL→fxs→CA1→DG from trajectory bundle I, TH→CA1→DG from trajectory bundle II, and VL→I→(V, VIa, VIb) from trajectory bundle III.

VL→fxs→CA1→DG from trajectory bundle I could represent the mechanism of how hematoma stimulation in the lateral ventricle directly induces hippocampal activities and cognitive dysfunction.

TH→CA1→DG from trajectory bundle II might represent the transmission of multiple signals from the lateral ventricle and signal integration *via* the hypothalamus to the hippocampus, which might be a relatively long-term effect of hematoma stimulation.

The changes in VL→I→(V, VIa, VIb) from trajectory bundle III might reflect the acute and direct effect of hematoma stimulation on the primary cortex.

Next, we carried out a union operation for the top 30 planar up/downregulated transition genes for the same subtrajectory in different plane sections at the same time to obtain the top 30 3D up/downregulated transition genes (**SR3 Table 2**) for each 3D subtrajectory by inputting **SR3 Table 1**. When we performed a union operation for the top 30 planar up/downregulated transition genes, if different plane sections had the same gene with a different sign (up- or downregulated), we chose the gene whose absolute value was the greatest.

Here, **Figures 3C–E** are examples of the top 30 3D up/downregulated transition genes for each subtrajectory in VL→fxs→CA1→DG from trajectory bundle I, TH→CA1→DG from trajectory bundle II, and VL→I→(V, VIa, VIb) from trajectory bundle III.

### 3.4 Cell subtype analysis

As discussed in the **Cell subtype analysis** section, we chose VL→fxs→CA1→DG from trajectory bundle I, TH→CA1→DG from trajectory bundle II, and VL→I→(V, VIa,VIb) from trajectory bundle III for further analysis.

**Figure 4:**
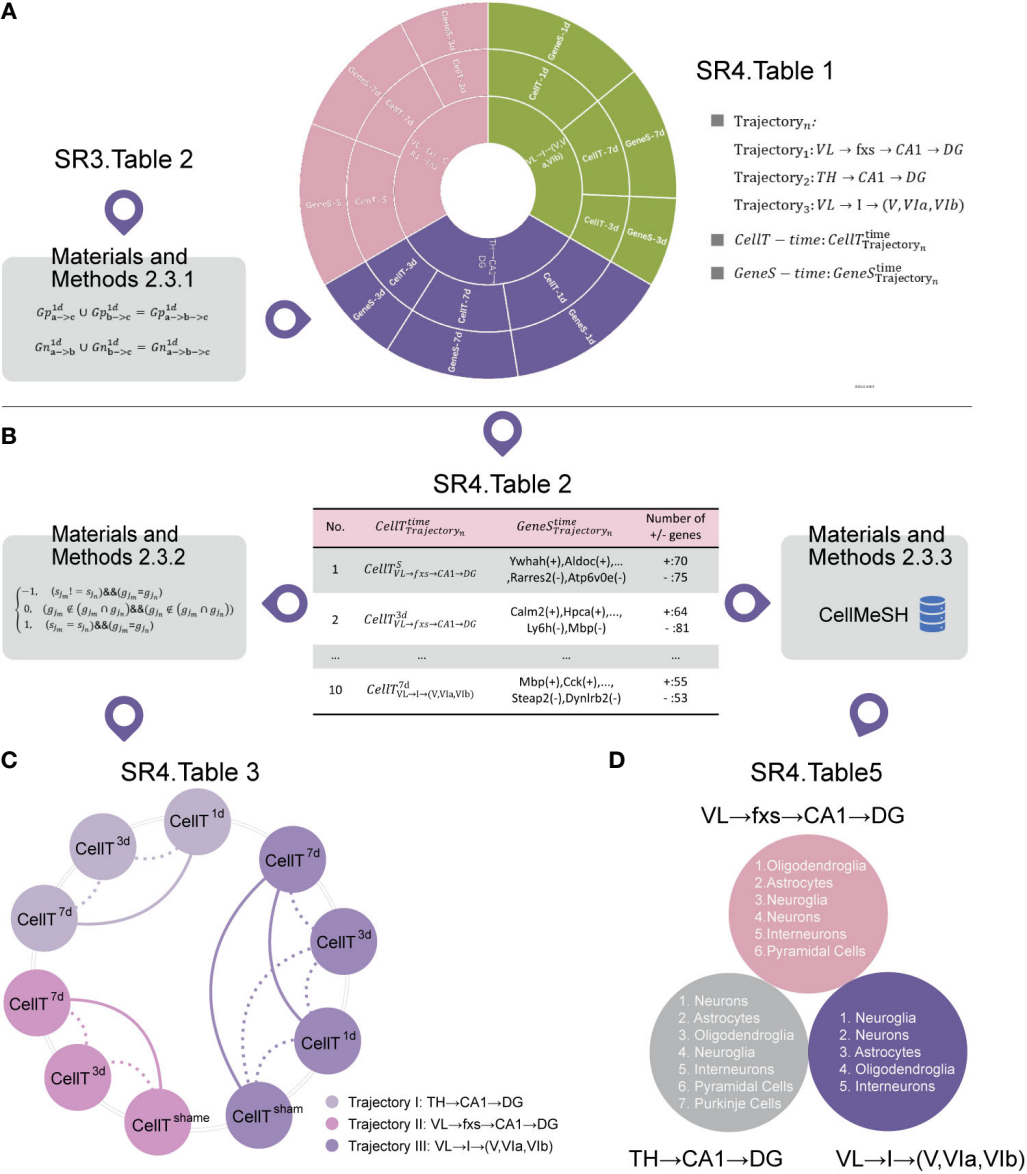
Cell subtype analysis. **(A)** The cell subtypes corresponding to the selected trajectory at different time points. **(B)** The marker gene sets of cell subtypes corresponding to the selected trajectory at different time points (**SR4 Table 2**). **(C)** Similarity analysis of cell subtypes at different time points within the same trajectory (**SR4 Table 3**). Among them, the thick line represents a high similarity between two time points, and the thin line represents a low similarity between two time points. **(D)** The cell types shared by our identified cell subtypes at different times within the same trajectory (**SR4 Table 5**).

Initially, we employed our developed algorithm (**SM Tables 4, 5**) to identify cell subtypes in the selected trajectory at different times (**Figure 4A** (**SR4 Table 1**)) by inputting the top 30 up/downregulated 3D transition genes for each 3D subtrajectory (**Figure 4A** (**SR3 Table 2**)), the marker gene sets of which are listed in **Figure 4B** (**SR4 Table 2**). This process is detailed in the cell subtype identification section of the Methods.

We then built a similarity algorithm (**SM Tables 4, 6**) to describe the similarity between cell subtypes within the same trajectory at different times (**Figure 4C** (**SR4 Table 3**)) by inputting the marker gene sets (**Figure 4B** (**SR4 Table 2**)). This process is detailed in the similarity algorithm for cell subtypes in the Methods section.

**Figure 4C** shows that the degree of cell subtype similarity between the third day and the seventh day is small for each selected trajectory. Additionally, the degree of cell subtype similarity between the sham group and the third day are both small, but the degree of cell subtype similarity between the sham group and the seventh day are both great for VL → fxs → CA1 → DG and VL → I→ (V, VIa, VIb).

These findings indicate a pattern in which not only are the cell subtypes similar for VL → fxs → CA1 → DG, TH → CA1 → DG, and VL → I → (V, VIa, VIb) after IVH stimulation, but these cell subtypes are also different from the cell subtypes of the sham group.

It should be noted that we do not have a cell subtype for the first day or the sham group for VL → fxs → CA1 → DG and TH → CA1 → DG, respectively. Additionally, **Figure 4C** (**SR4 Table 3**) shows that the pattern is not as obvious for VL → I → (V, VIa, VIb).

Finally, we found cell types (**Figure 4D** (**SR4 Table 4**)) from a commonly used public single-cell sequencing database (CellMeSH (23)) that are similar to our identified cell subtypes (**Figure 4A** (**SR4 Table 1**)) as described in the Methods section. Additionally, **Figure 4D** (**SR4 Table 5**) describes the cell types that are most similar to our identified cell subtypes at different times within the same trajectory.

### 3.5 Cell−cell communication strength analysis

**Figure 5:**
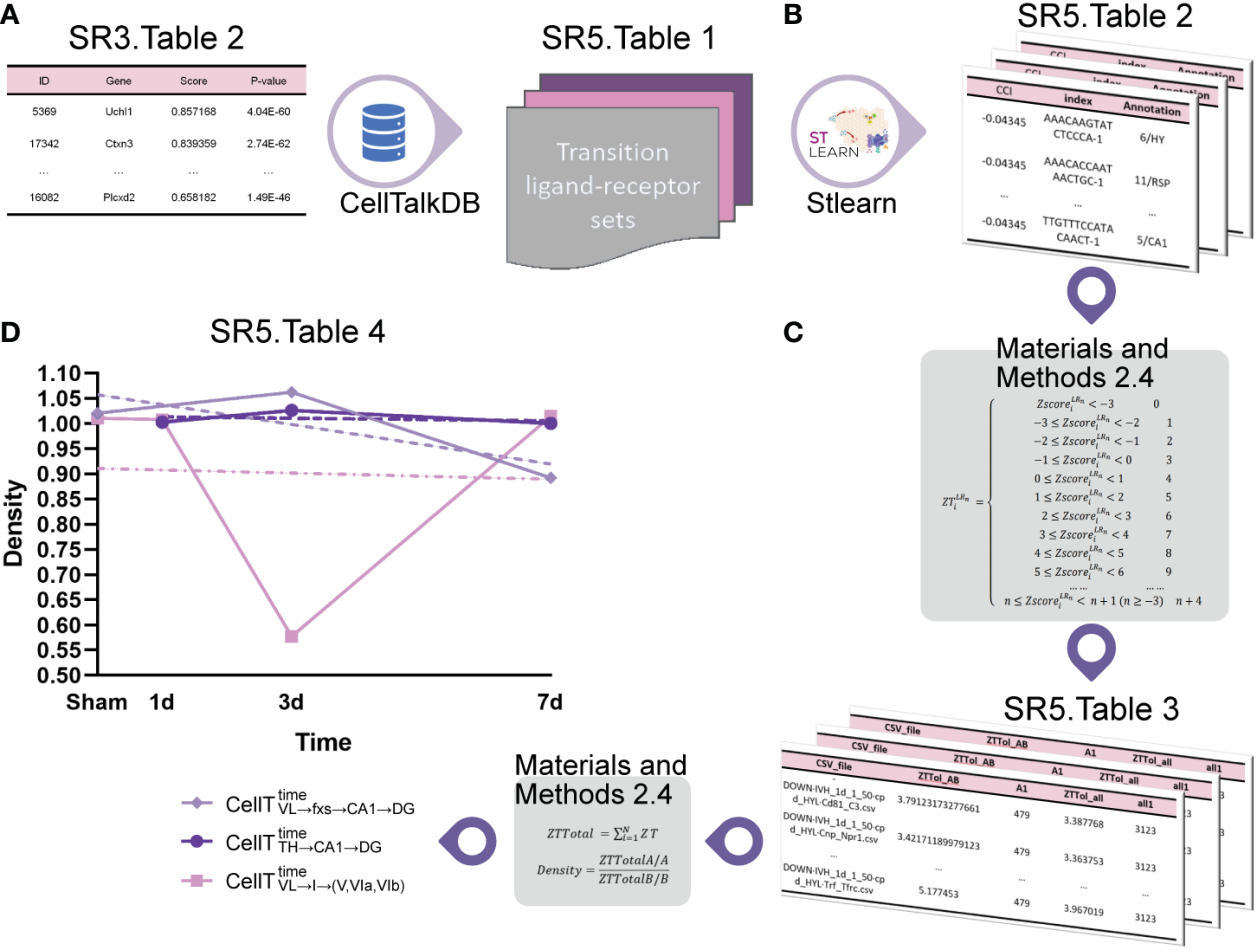
Cell−cell communication strength analysis. **(A)** The transition ligand–receptor sets. **(B)** The interaction intensity value $Z{\text{score}}_{i}^{{\text{LR}}_{n}}$ . **(C)** The discrete interaction intensity value $ZT_{i}^{{\text{LR}}_{n}}$ . **(D)** The cell−cell communication strength (density).

Initially, we employed the CellTalkDB database (34) to locate ligand–receptor pairs by inputting the up- and downregulated transition gene sets (**Figure 5A** (**SR3 Table 2**)). **Figure 5A** (**SR5 Table 1**) shows the up/downregulated transition ligand–receptor (LR*_n_*) pairs.

Next, we employed stLearn software (12) to analyze their interaction intensity by using **Figure 5A** (**SR5 Table 1**) as the input. **Figure 5B** (**SR5 Table 2**) shows the interaction intensity values, $Z{\text{score}}_{i}^{{\text{LR}}_{n}}$ , for the up/downregulated transition ligand–receptor (LR*_n_*) pairs. We then obtained the discrete interaction intensity $ZT_{i}^{{\text{LR}}_{n}}$ (**Figure 5C** (**SR5 Table 3**)) and the average Density^LR^ *^n^* values for each ligand–receptor pair in the cell subtypes (**Figure 5D** (**SR5 Table 4**)) by using the cell−cell communication analysis described in the Methods section.

**Figure 5D** shows that the density of $\text{Cell}T_{\text{VL}\to \text{fxs}\to \text{CA}1\to \text{DG}}^{\text{time}}$ increased from $\text{Cell}T_{\text{VL}\to \text{fxs}\to \text{CA}1\to \text{DG}}^{\text{Sham}}$ to $\text{Cell}T_{\text{VL}\to \text{fxs}\to \text{CA}1\to \text{DG}}^{3d}$ and decreased from $\text{Cell}T_{\text{VL}\to \text{fxs}\to \text{CA}1\to \text{DG}}^{3d}$ to $\text{Cell}T_{\text{VL}\to \text{fxs}\to \text{CA}1\to \text{DG}}^{7d}$ ; the density of $\text{Cell}T_{\text{TH}\to \text{CA}1\to \text{DG}}^{\text{time}}$ increased from $\text{Cell}T_{\text{TH}\to \text{CA}1\to \text{DG}}^{1d}$ to $\text{Cell}T_{\text{TH}\to \text{CA}1\to \text{DG}}^{3d}$ but decreased from $\text{Cell}T_{\text{TH}\to \text{CA}1\to \text{DG}}^{3d}$ to $\text{Cell}T_{\text{TH}\to \text{CA}1\to \text{DG}}^{7d}$ ; and the density of $\text{Cell}T_{\text{VL}\to \text{I}\to \left(\text{V},\text{VIa},\text{VIb}\right)}^{\text{time}}$ decreased from $\text{Cell}T_{\text{VL}\to \text{I}\to \left(\text{V},\text{VIa},\text{VIb}\right)}^{\text{sham}}$ to $\text{Cell}T_{\text{VL}\to \text{I}\to \left(\text{V},\text{VIa},\text{VIb}\right)}^{1d}$ and from $\text{Cell}T_{\text{VL}\to \text{I}\to \left(\text{V},\text{VIa},\text{VIb}\right)}^{1d}$ to $\text{Cell}T_{\text{VL}\to \text{I}\to \left(\text{V},\text{VIa},\text{VIb}\right)}^{3d}$ but increased from $\text{Cell}T_{\text{VL}\to \text{I}\to \left(\text{V},\text{VIa},\text{VIb}\right)}^{3d}$ to $\text{Cell}T_{\text{VL}\to \text{I}\to \left(\text{V},\text{VIa},\text{VIb}\right)}^{7d}$ .

Finally, the Kruskal−Wallis test (35) (**SR5 Table 5**) demonstrates that the density is significantly different among these cell subtypes in VL → fxs → CA1 → DG, TH → CA1 → DG, and VL → I → (V, VIa, VIb).

Therefore, we hypothesized that the cell−cell communication strength would greatly change on the third day after IVH stimulation and return to normal on the seventh day.

### 3.6 Pathway analysis

**Figure 6:**
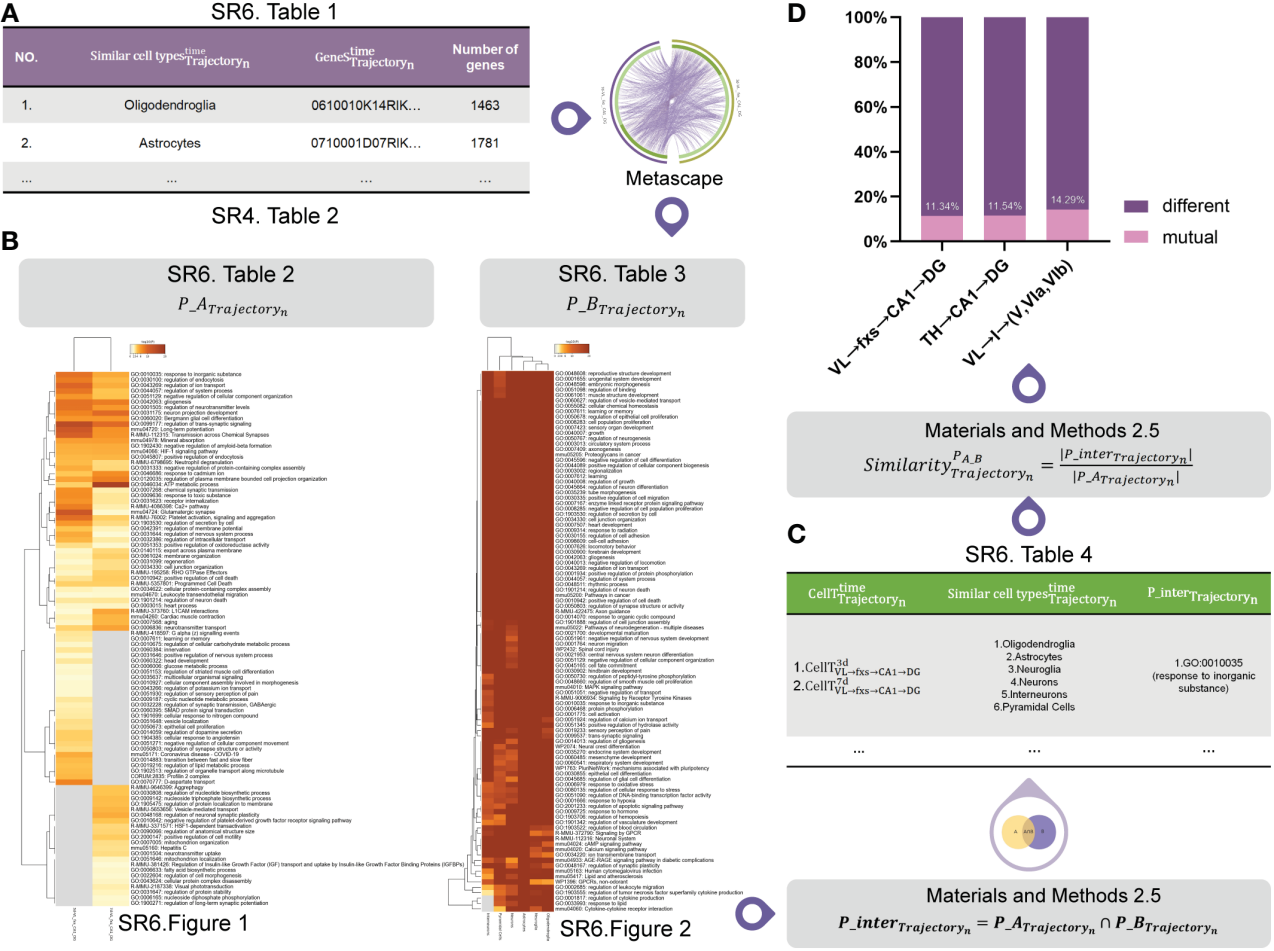
Pathway analysis. **(A)** The marker genes of similar cell types. **(B)** The mutual pathway sets for our identified cell subtypes *P*_*A*_Trajectory_*n*__ and similar cell types *P*_*B*_Trajectory_*n*__ . **(C)** The mutual pathway set *P*_*inter*_Trajectory_*n*__ between our identified cell subtypes *P*_*A*_Trajectory_*n*__ and similar cell types *P*_*B*_Trajectory_*n*__ . **(D)** The similarity between *P*_*A*_Trajectory_*n*__ and *P*_*B*_Trajectory_*n*__ .

First, we employed CellMeSH (23) to identify the genes associated with similar cell types (**Figure 6A** (**SR6 Table 1**)) by inputting similar cell types (**Figure 6A** (**SR4 Table 5**)).

Next, using **Figure 6A** (**SR6 Table 1**) and marker genes of our identified cell subtypes (**Figure 6A** (**SR4 Table 2**)) as the input, we employed Metascape software (15) to carry out pathway analysis for each of them. **Figure 6B** shows mutual pathway sets for our identified cell subtypes *P*_*A*_Trajectory_*n*__ (**Figure 6B** (**SR6 Table 2** and **SR6 Figure 1**)) and similar cell types *P*_*B*_Trajectory_*n*__ (**Figure 6B** (**SR6 Table 3** and **SR6 Figure 2**)).

**Figure 6C** (**SR6 Table 4**) shows the mutual pathway set *P*_*inter*_Trajectory_*n*__ between our identified cell subtypes *P*_*A*_Trajectory_*n*__ and similar cell types *P*_*B*_Trajectory_*n*__ , the process of which is detailed in the signaling pathway analysis section of the Methods.

**Figure 6D** shows that our identified cell subtypes and their similar cell types in VL → fxs → CA1 → DG, TH → CA1 → DG, and VL → I → (V, VIa, VIb) have 11, nine, and nine mutual pathways, respectively. Since they have mutual pathways (**Figure 6C** (**SR6 Table 4**)) and the proportion of these mutual pathways remained stable for each trajectory, we hypothesized that our identified cell subtypes and their similar cell types found using CellMeSH software (23) should be at the same molecular level.

## 4 Discussion

In the present study, we established an IVH mouse model by generating a hematoma in the lateral ventricle of the brain as primary stimulation for the ipsilateral hemisphere in mice. We then developed spatial transcriptome sequencing-based bioinformatic algorithms to investigate the 3D spatial brain regions affected by the primary stimulation of the hematoma in the ipsilateral ventricle after IVH (**Figure 1**). Investigations using this model have resulted in the following important findings: (1) the discovery of three 3D global pseudospace-time trajectories (**Figure 2**); (2) the identification of the cell subtypes within these trajectories at different times after IVH stimulation (**Figure 3**) and the elucidation of a similar pattern for these cell types within each 3D global pseudospace-time trajectory (**Figure 4**); (3) the observation that the cell−cell communication strength greatly changes after IVH stimulation and returns to a normal state later (**Figure 5**); and (4) the notion that our identified cell subtypes and their similar cell types found in the single-cell sequencing database (CellMeSH (23)) should be similar to each other at the molecular level (**Figure 6**).

**SM Figure 1** indicates that 25 μl of autologous blood was injected into the contralateral ventricle to establish the IVH model used in this study, which exhibited obvious hematoma drainage across the interventricular foramen and deposition in the ipsilateral ventricle but without direct damage to the ependymal barrier in the ventricles. We suggest that this hematoma establishment method produces the ideal model to investigate the secondary neurological dysfunction derived from hematoma stimulation after IVH. The 3D global pseudospace-time trajectories (**Figures 2E–G**) indicated that hematoma stimulation in the lateral ventricle mainly affected the ipsilateral cortex and hippocampus. A recent analysis of the functional connectivity of the ipsilateral cortex indicated that IVH in infants born moderately preterm and later preterm was associated with the frontoparietal operculum and orbitofrontal cortex, which are related to language and cognition during development (36). In addition, low-grade IVH after germinal matrix hemorrhage in preterm neonates was reported to cause lower cerebral blood flow in posterior cortical and subcortical gray matter regions (37). Trajectory bundle III encompasses changes directly from the lateral ventricle to the ipsilateral cortex, suggesting the regional vulnerability of these brain structures. However, intraventricular extension after intracerebral hemorrhage might not be associated with dysphagia, even with a space-occupying effect and midline shift (38). As **Figure 4C** (**SR4 Table 3**) illustrates, we revealed a pattern in which not only are the cell subtypes after IVH stimulation similar for VL → fxs → CA1 → DG, TH → CA1 → DG, and VL → I → (V, VIa, VIb), but these cell subtypes are also different from the cell subtypes of the sham group. However, **Figure 4C** (**SR4 Table 3**) demonstrates that the pattern is not as obvious for VL → I → (V, VIa, VIb) compared to that of VL → fxs → CA1 → DG and TH → CA1 → DG. We explain this phenomenon as follows: (1) motor dysfunction usually occurs in most clinical patients with isolated IVH, and (2) our data show that lateral ventricle stimulation has only a transient effect on the ipsilateral cortex.

However, since most patients with IVH are in a supine resting state after onset, there is no clinical evidence to accurately describe the severity of motor dysfunction in its acute phase, and the effects of hematoma stimulation largely returned to normal after 7 days in model mice.

In addition to the limited evidence of hematoma stimulation in the lateral ventricle affecting the cortex, we also illustrated two major 3D global pseudospace-time trajectory bundles involving the hippocampus. Trajectory bundle I (**Figures 4C** (**SR2 Figures 3.1A, B**, **3.2B**, **3.3B**)) is a direct neuronal circuit from the lateral ventricle to the hippocampus, which, from our understanding, is closely associated with white matter lesions after intraventricular extension in spontaneous intracerebral hemorrhage patients (39). Trajectory bundle II (**Figures 4C** (**SR2 Figures 3.1C**, **3.2C**, **3.3C**)) is a novel integrated signaling circuit from the hypothalamus to the hippocampus that flexibly modulates long-term potentiation (40) and several factors of cognition (41). In addition, corpus callosum injury is also reported to strongly correlate with the severity of IVH (42) and serves as a prognostic marker for poor outcomes after brain trauma. Consistent with our previous rodent experiment, perihematomal tissue injury and neurocognitive deficits were reported in intracerebral hemorrhage with ventricular extension (43), and blood metabolites such as iron (44), oxyhemoglobin (45), and thrombin (45, 46) are well considered in this pathophysiological process. Combined with the negative results of the CLEAR III clinical trial, our data in **Figure 4C** indicated that the presence of a hematoma rapidly stimulates the direct neuronal circuit from the lateral ventricle to the hippocampus, and clearing the blood clots in the ventricle might not successfully remove these stimulations in the cerebrospinal fluid. Targeting subsequent secondary brain injuries due to hematoma stimulation in the ventricles might be a promising therapeutic strategy for IVH patients. **Figure 4D** (**SR4 Table 5**) illustrates the most affected or activated cell types by our identified cell subtypes (**Figure 4B** (**SR4 Table 2**)), which clearly correspond to the potential pathophysiological changes in these main trajectories/neural circuits after IVH stimulation.

For VL→fxs→CA1→DG from trajectory bundle I, which might be tightly associated with white matter lesions, as we discussed above, the most activated cell type is oligodendroglia, which is consistent with its being widely understood as the main participant in white matter and cognitive functions. Further pathway analysis indicated intense metabolic changes and synaptic activity, as well as gliogenesis and neural cell death in this trajectory. The cell−cell communication strength analysis exhibited strong cell–cell communication after IVH stimulation, which substantially weakened, as shown in **Figure 5D**. During the post-IVH period, 11 mutual pathways (**Figure 6D**) reflected the core pathophysiological changes, and these pathways may be potential clues and therapeutic targets for further preclinical study.

Additionally, in TH→CA1→DG from trajectory bundle II, it seems that neuronal cells are the main participants and cell proliferation is the pathway with the most substantial changes, which is supported by a recent study (47) that suggested hypothalamic circuits could regulate memory by modulating adult hippocampal neurogenesis.

However, pathway analysis (**SR6 Figure 1.3**) of VL→I→(V, VIa, VIb) from trajectory bundle III indicated an acute response to hypoxia and oxidative stress, which is consistent with the significant suppression of cell−cell communication strength within this trajectory on the third day after IVH stimulation (**Figure 5D**). Nevertheless, the communication strength recovered to a normal level on the seventh day after IVH stimulation, which might be an explanation for the short-term motor dysfunction in IVH patients and is worthy of clinical attention in the future.

Our proposed bioinformatics analysis workflow has the following innovations and limitations: First, we developed a 3D global pseudospace-time trajectory reconstruction algorithm that cannot only investigate the genetic changes in 3D global pseudospace-time trajectories but also identify cell subtypes within these trajectories at different times. However, due to the limited sections and the conflict between planner trajectories, this algorithm cannot obtain high accuracy.

Second, because our proposed similarity algorithm considers the up/downregulation of marker genes of cell subtypes, we can accurately compute the similarity between cell subtypes at different times on the same trajectory. However, since spatial transcriptome data only provide discrete up/downregulation data, the accuracy of our similarity algorithm is limited.

Third, our proposed cell−cell communication intensity density algorithm can uniformly measure cell−cell communication intensity for multiple spatial transcriptome sections at different times for each ligand−receptor pair within a cell subtype compared to a previous method (12) that analyzed cell−cell communication intensity for a single section. However, since we do not have an automatic algorithm to match cell subtype ligand–receptor pairs in the CellTalkDB database (34), it was time-consuming for us to process the cell subtypes that had many paired ligands.

In conclusion, we innovated a bioinformatics algorithm to discover three main 3D global pseudospace-time trajectory groups that represent the main neural circuits from the lateral ventricle to the hippocampus and primary cortex affected by experimental IVH stimulation. Further analysis indicated a rapid response in the primary cortex as well as a direct and integrated effect on the hippocampus after IVH stimulation. To the best of our knowledge, this is the first study to investigate secondary brain injury after IVH stimulation by using spatial transcriptome sequencing and bioinformatics analysis, but verification and further investigation by basic neuroscience and translational interventions are still needed. These data provide helpful information for elucidating the pathophysiological mechanism for the occurrence of IVH in patients after acute brain injury, as well as algorithm strategies for similar studies in the future. The algorithm strategies used in this study are searchable *via* a web service (www.combio-lezhang.online/3dstivh/home).

## Data availability statement

The datasets presented in this study can be found in online repositories. The names of the repository/repositories and accession number(s) can be found below: https://www.ncbi.nlm.nih.gov/geo/, GSE214349.

## Ethics statement

The animal study was reviewed and approved by Laboratory Animal Welfare and Ethics Committee of Third Military Medical University (AMUWEC2020762).

## Author contributions

LZ and YC conceptualized and designed the experiments and supervised the research. XR, SH, and YC performed animal experiments. LZ, JB, GW, WS, YY, JH, and YC acquired and analyzed the data. LZ, JB, GW, WS, HF, RC, YZ, and YC interpreted the data. LZ, JB, WS, YZ, and YC drafted the manuscript. All authors have read and approved the current version of the manuscript.
