# Supplementary material for: Discovering hematoma-stimulated circuits for secondary brain injury after intraventricular hemorrhage by spatial transcriptome analysis
Source: Front Immunol. 2023 Feb 7;14:1123652. doi: 10.3389/fimmu.2023.1123652 (PMC9941151; doi:10.3389/fimmu.2023.1123652)
Supplement: Supplementary file 1 [file DataSheet_1.pdf]

Supplementary Materials

This Supplementary Materials consists of two sections. One is **Supplementary of Results (SR)** and the other is **Supplementary of Methods (SM)**.

I. Supplementary of Results (SR)

This Supplementary of Results section consists of 6 supplementary sections, which is listed as below.

1. Supplementary of Result 1 (SR1) (Page 2-12)
2. Supplementary of Result 2 (SR2) (Page 13-20)
3. Supplementary of Result 3 (SR3) (Page 21)
4. Supplementary of Result 4 (SR4) (Page 21)
5. Supplementary of Result 5 (SR5) (Page 22-24)
6. Supplementary of Result 6 (SR6) (Page 24-31)

II. Supplementary of Methods (SM)

This Supplementary of Methods section consists of 11 supplementary files, which is listed as below.

Figure1 IVH model (Page 32)

Table 1 IVH sample grouping, library patching strategy and permeabilization time（min）(Page 33)

Table 2 Parameter Definition for 3D global Pseudo-space-time trajectory reconstruction algorithm (Page 34-36)

Table 3 3D global Pseudo-space-time trajectory reconstruction algorithm (Page 37-38)

Table 4 Parameter Definition for the algorithm to identify a cell subtype and Similarity algorithm for cell subtypes (Page 39-40)

Table 5 The algorithm to identify a cell subtype (Page 41)

Table 6 Similarity algorithm for cell subtypes (Page 42)

Table 7 Parameter Definition for Cell-cell communication strength (Density) algorithm (Page 43-44)

Table 8 Cell-cell communication strength (Density) algorithm (Page 45)

Table 9 Parameter Definition for Similarity algorithm for mutual pathway sets (Page 46)

Table 10 Similarity algorithm for mutual pathway sets (Page 47)

I. Supplementary of Results (SR)

This supplementary of results section consists of 6 supplementary sections, which is listed as below.

1. Supplementary of Result 1 (SR1)

Figure 1 H&E (Hematoxylin and Eosin stain) slices of mouse brain after IVH

1.1 H&E (Hematoxylin and Eosin stain) slices of mouse brain after IVH.

(A) IVH_C1_50. **(B)** IVH_C1_110. **(C)** IVH_S1_50. **(D)** IVH_S1_80. **(E)** IVH_S1_110. **(F)** IVH_1d_20. It is noted that **(A)** IVH_C1_50 represents the H&E slices of mouse brain at position 50 for control group.

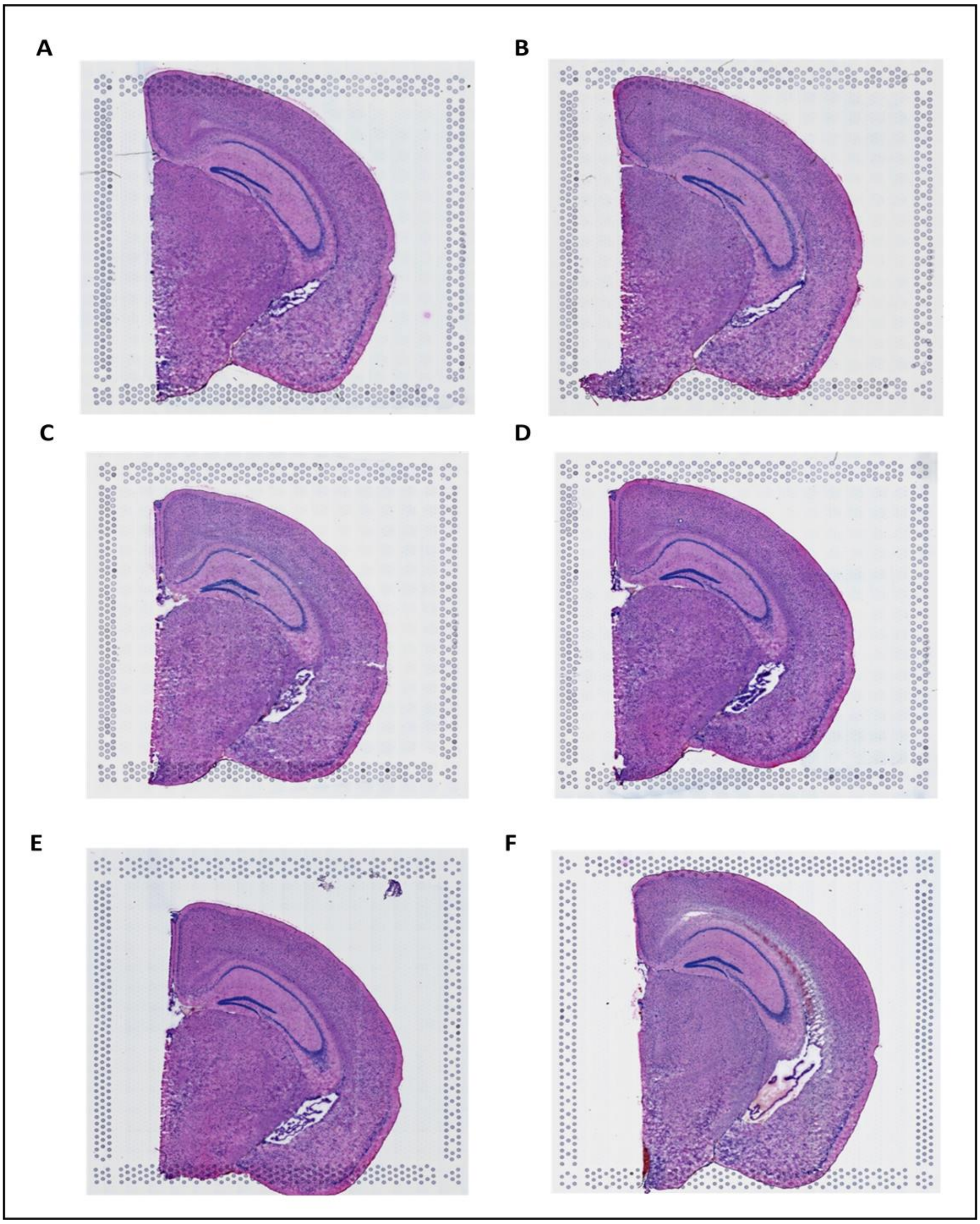

1.2 H&E (Hematoxylin and Eosin stain) slices of mouse brain after IVH. **(A)** IVH_1d_50. **(B)** IVH_1d_80. **(C)** IVH_1d_110. **(D)** IVH_1d_130. **(E)** IVH_1d_150. **(F)** IVH_3d_20.

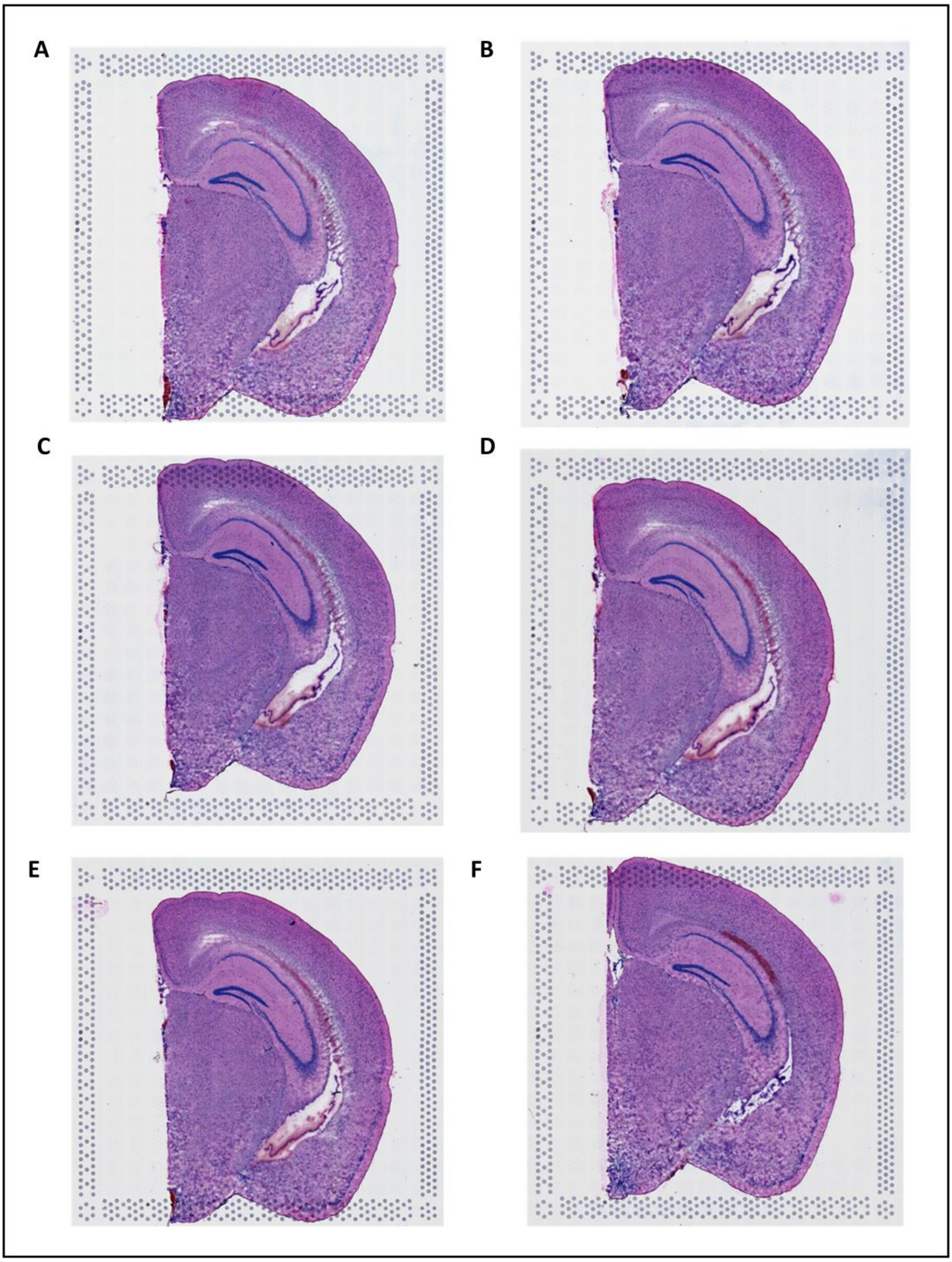

1.3 H&E (Hematoxylin and Eosin stain) slices of mouse brain after IVH. **(A)** IVH_3d_50. **(B)** IVH_3d_110. **(C)** IVH_3d_130. **(D)** IVH_3d_150. **(E)** IVH_7d_50. **(F)** IVH_7d_80. **(G)** IVH_7d_110.

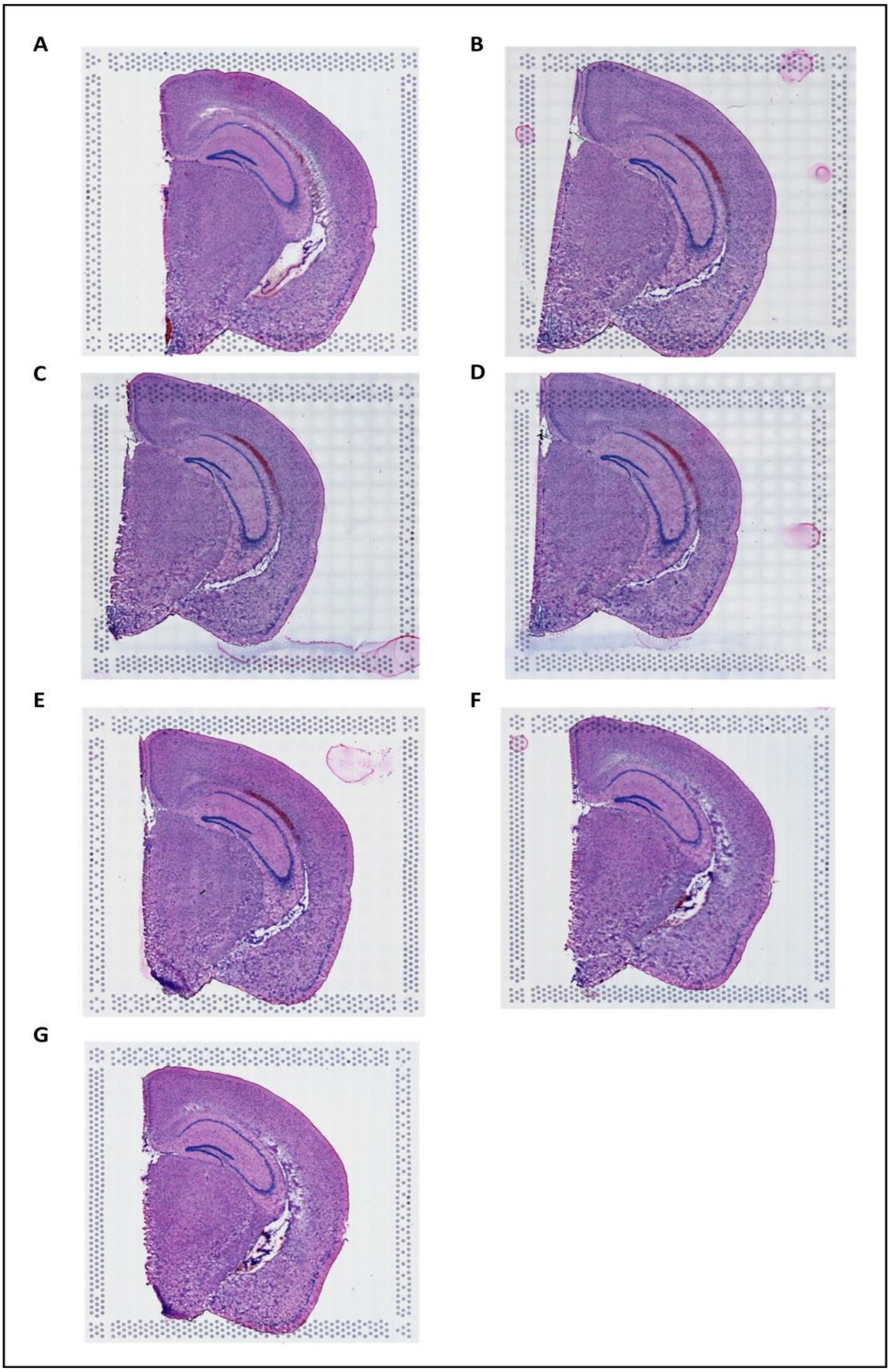

Figure 2 Cell type clustering for mouse brain

2.1 Cell type clustering for mouse brain. **(A)** IVH_C1_50. **(B)** IVH_C1_110. **(C)** IVH_S1_50. **(D)** IVH_S1_80. **(E)** IVH_S1_110. **(F)** IVH_1d_1_20. **(G)** IVH_1d_1_50. **(H)** IVH_1d_1_80. **(I)** IVH_1d_1_110.

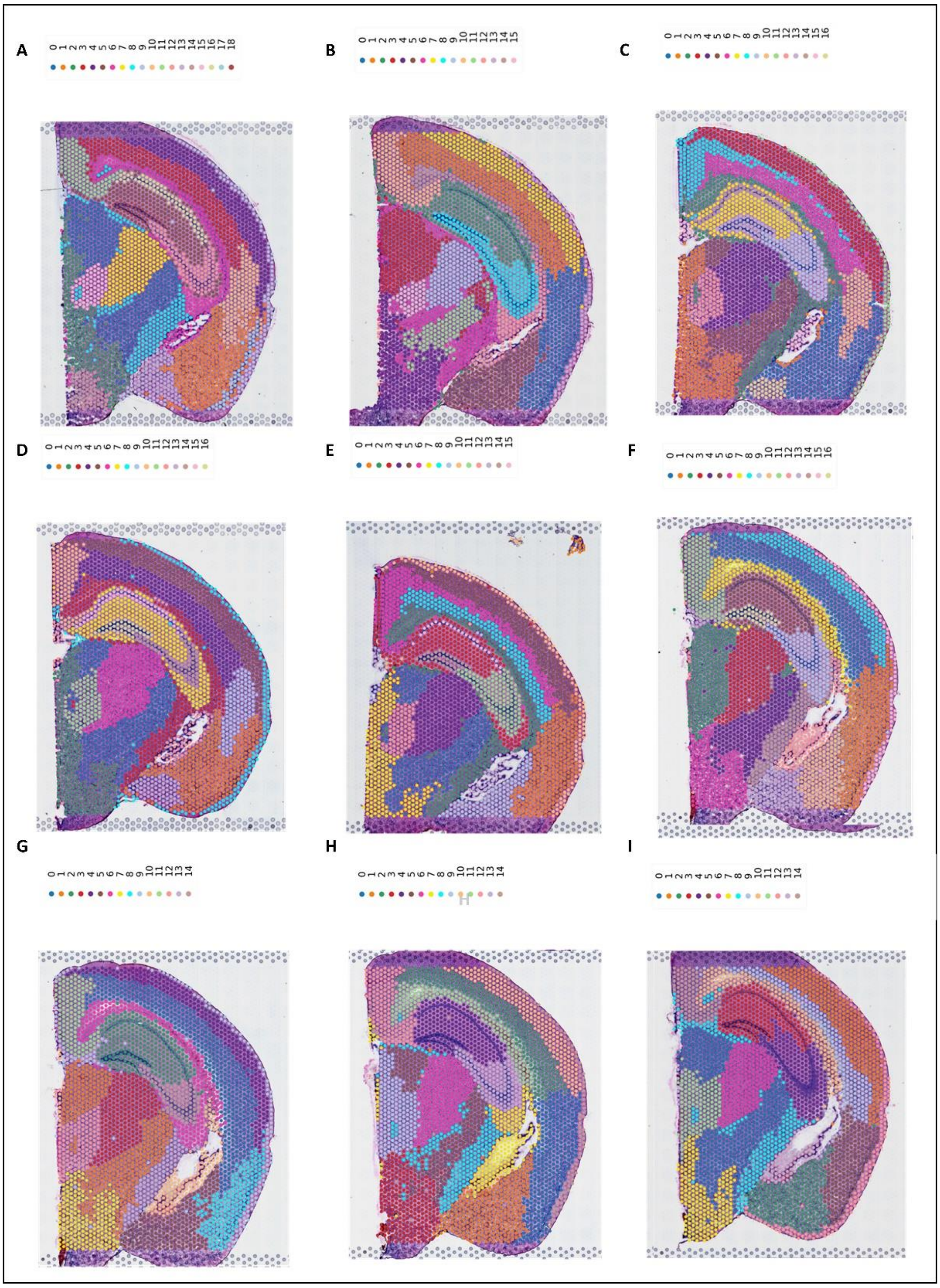

2.2 Cell type clustering for mouse brain. **(A)** IVH_1d_1_130. **(B)** IVH_1d_1_150. **(C)** IVH_3d_3_20. **(D)** IVH_3d_3_50. **(E)** IVH_3d_3_110. **(F)** IVH_3d_3_130. **(G)** IVH_3d_3_150. **(H)** IVH_7d_2_50. **(I)** IVH_7d_2_80. **(J)** IVH_7d_2_110.

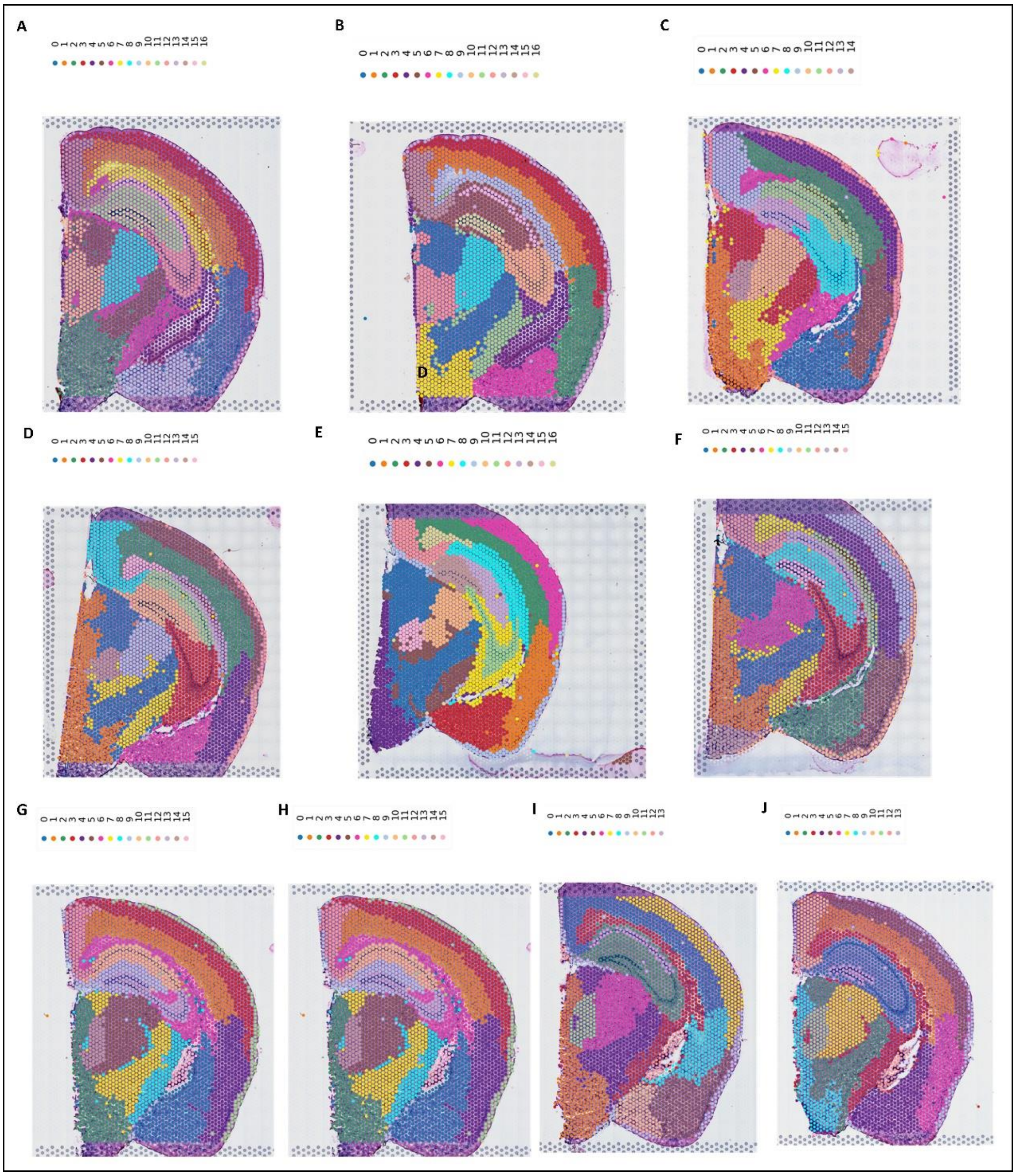

Figure 3 The annotation atlas of cell type clusters for mouse brain

3.1 The annotation atlas of cell type clusters for mouse brain. **(A)** IVH_C1_50. **(B)** IVH_C1_110. **(C)** IVH_S1_50. **(D)** IVH_S1_80.

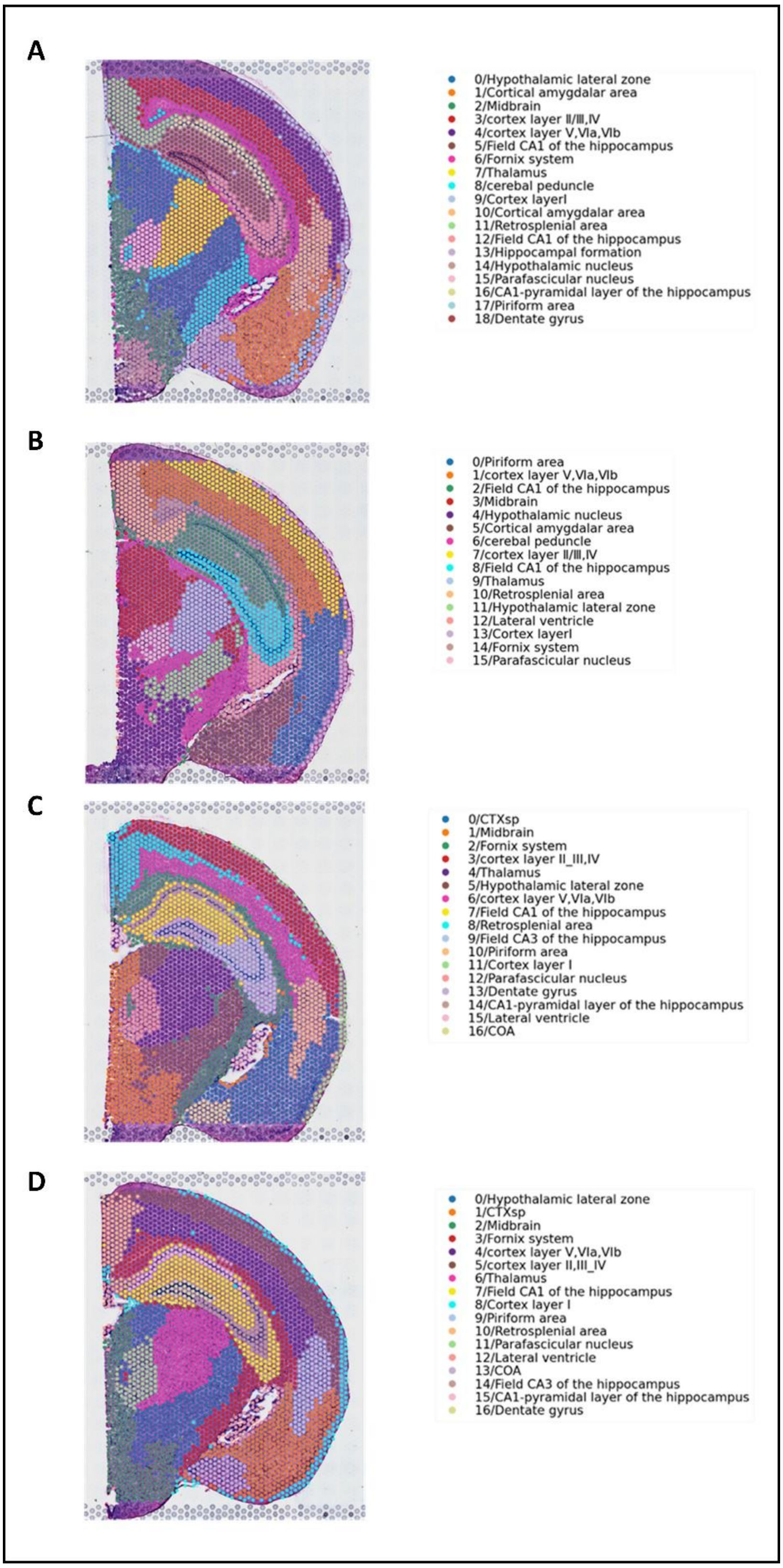

3.2 The annotation atlas of cell type clusters for mouse brain. **(A)** IVH_S1_110. **(B)** IVH_1d_1_20. **(C)** IVH_1d_1_50. **(D)** IVH_1d_1_80.

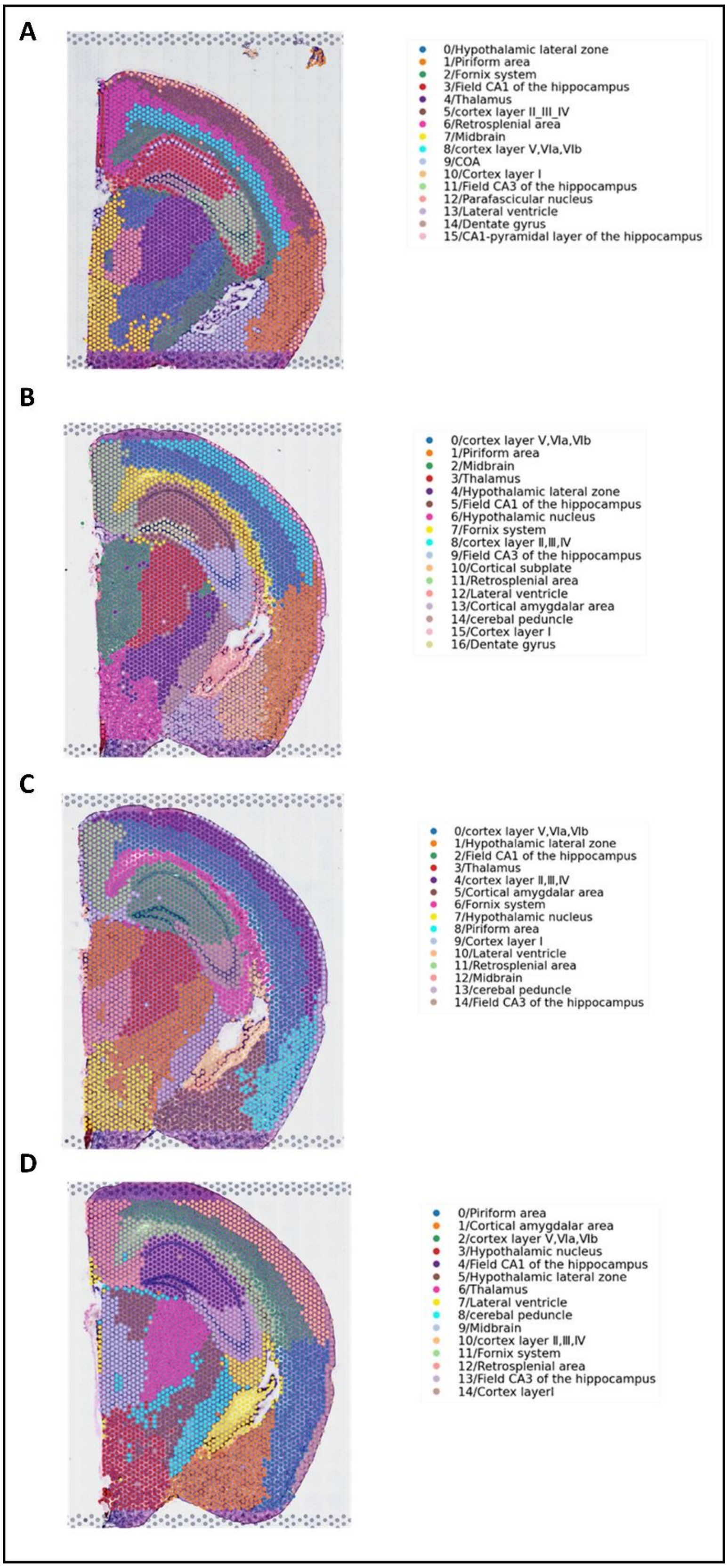

3.3 The annotation atlas of cell type clusters for mouse brain. **(A)** IVH_1d_1_110. **(B)** IVH_1d_1_130. **(C)** IVH_1d_1_150. **(D)** IVH_3d_3_20.

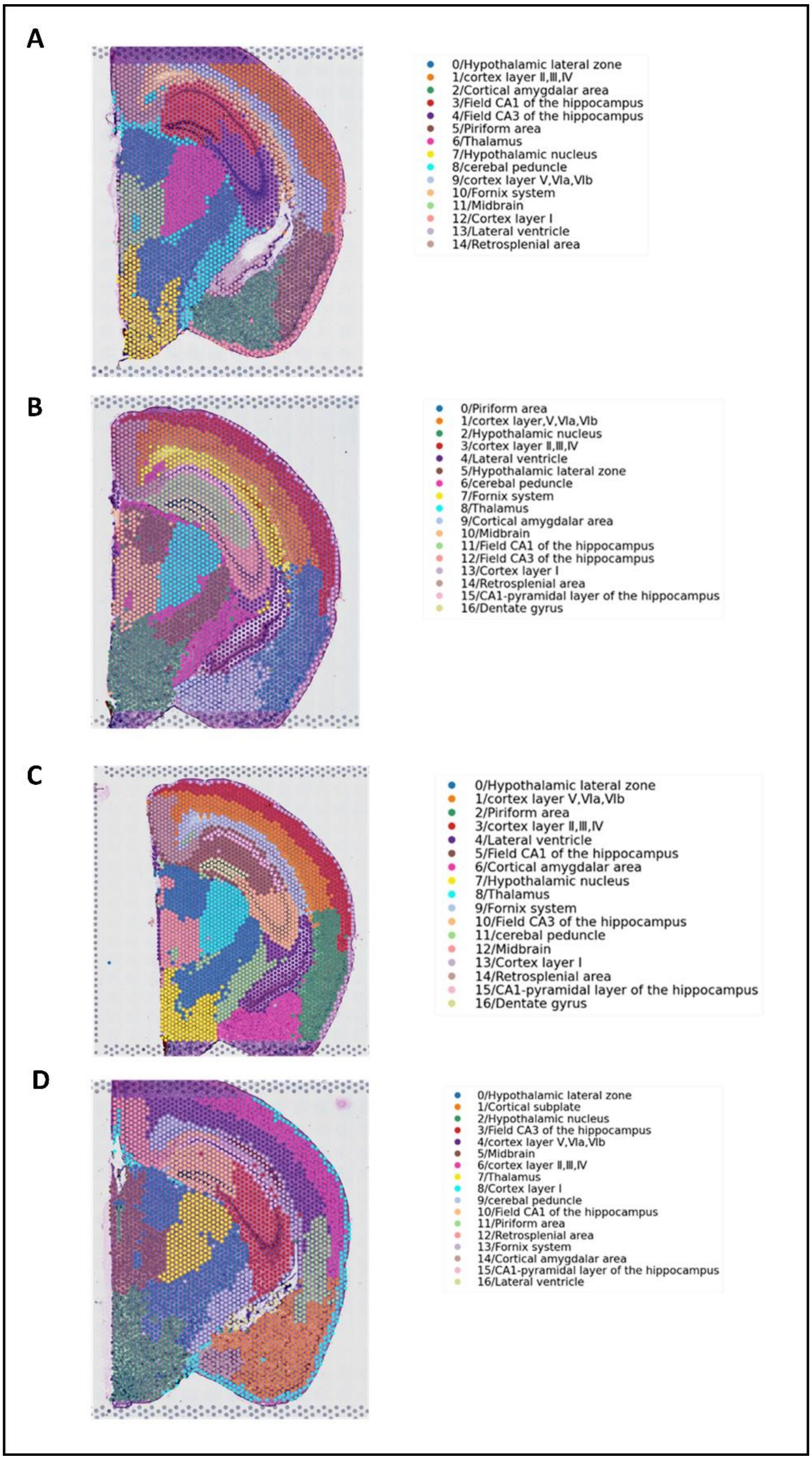

3.4 The annotation atlas of cell type clusters for mouse brain. **(A)** IVH_3d_3_50. **(B)** IVH_3d_3_110. **(C)** IVH_3d_3_130. **(D)** IVH_3d_3_150.

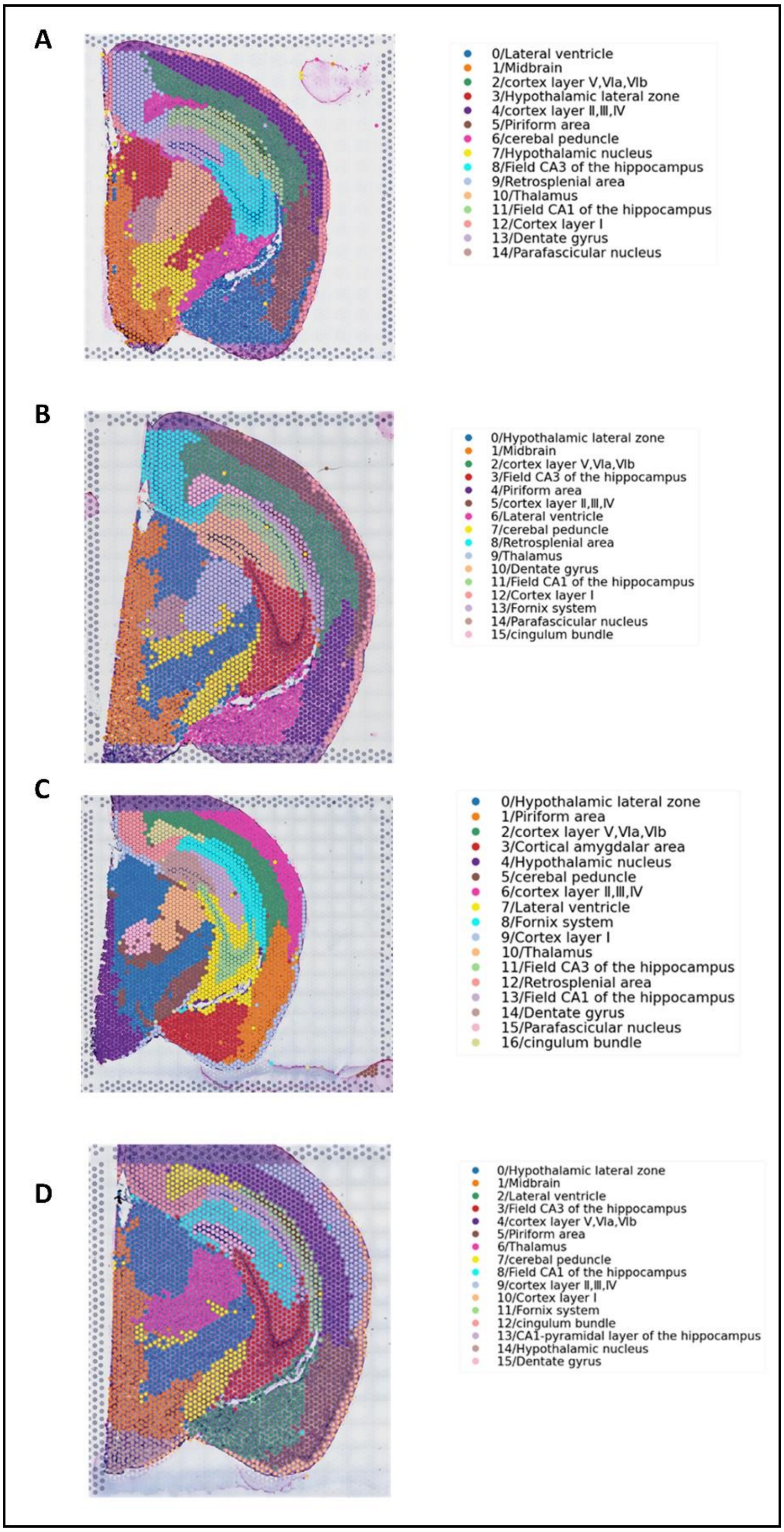

3.5 The annotation atlas of cell type clusters for mouse brain. **(A)** IVH_7d_2_50. **(B)** IVH_7d_2_80. **(C)** IVH_7d_2_110.

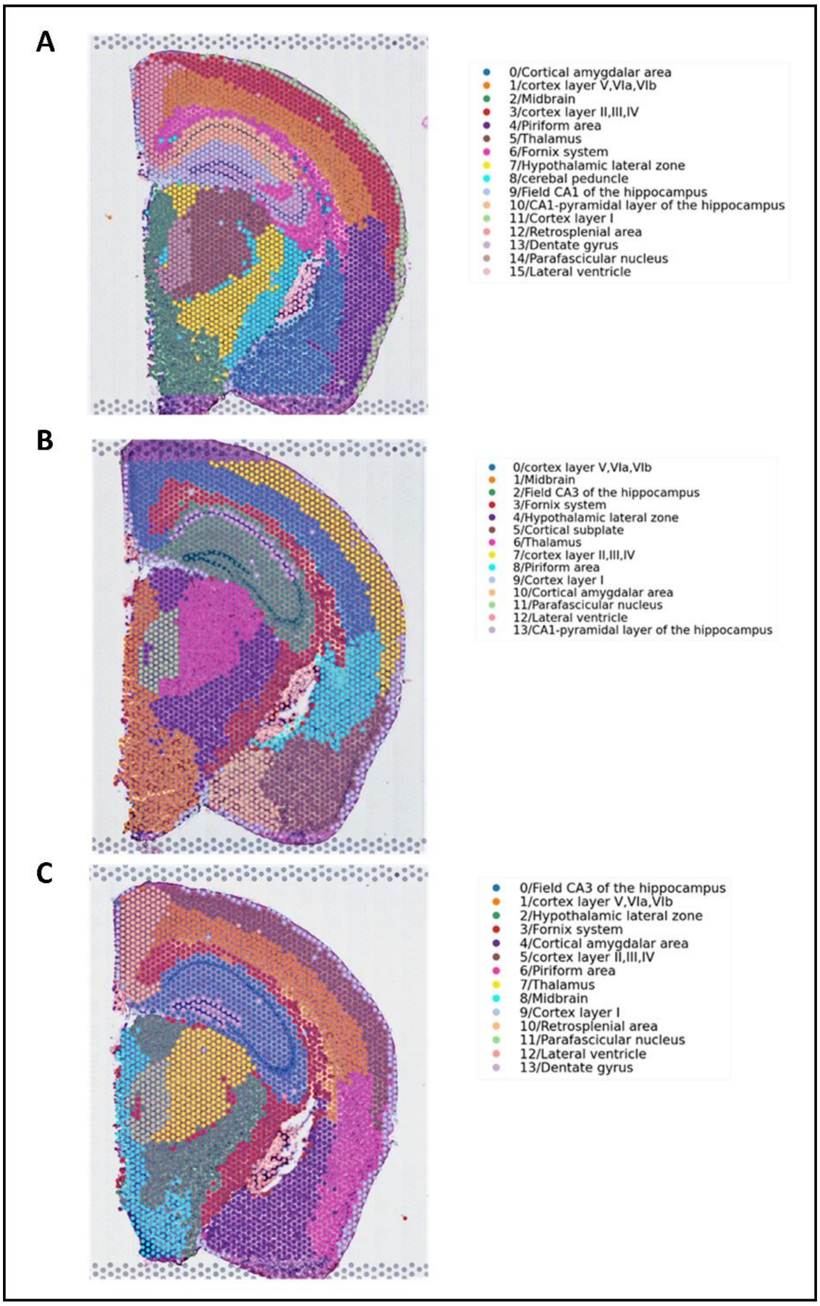

Table 1 The tissue position and spatial gene expression of mouse brain after IVH https://github.com/JiayidaerBadai/Spatial-transcriptome.git

a. IVH_C1_50
b. IVH_C1_110
c. IVH_S1_50
d. IVH_S1_80
e. IVH_S1_110
f. IVH_1d_1_20
g. IVH_1d_1_50
h. IVH_1d_1_80
i. IVH_1d_1_110
j. IVH_1d_1_130
k. IVH_1d_1_150
l. IVH_3d_3_20
m. IVH_3d_3_50
n. IVH_3d_3_110
o. IVH_3d_3_130
p. IVH_3d_3_150
q. IVH_7d_2_50
r. IVH_7d_2_80
s. IVH_7d_2_110

2. Supplementary of Result 2 (SR2)

Figure 1 The planar diffusion pseudotime plots

1.1 The planar diffusion pseudotime plots. **(A)** IVH_S1_50. **(B)** IVH_S1_80. **(C)** IVH_S1_110. **(D)** IVH_1d_1_20. **(E)** IVH_1d_1_50. **(F)** IVH_1d_1_80. **(G)** IVH_1d_1_110. **(H)** IVH_1d_1_130. **(I)** IVH_1d_1_150.

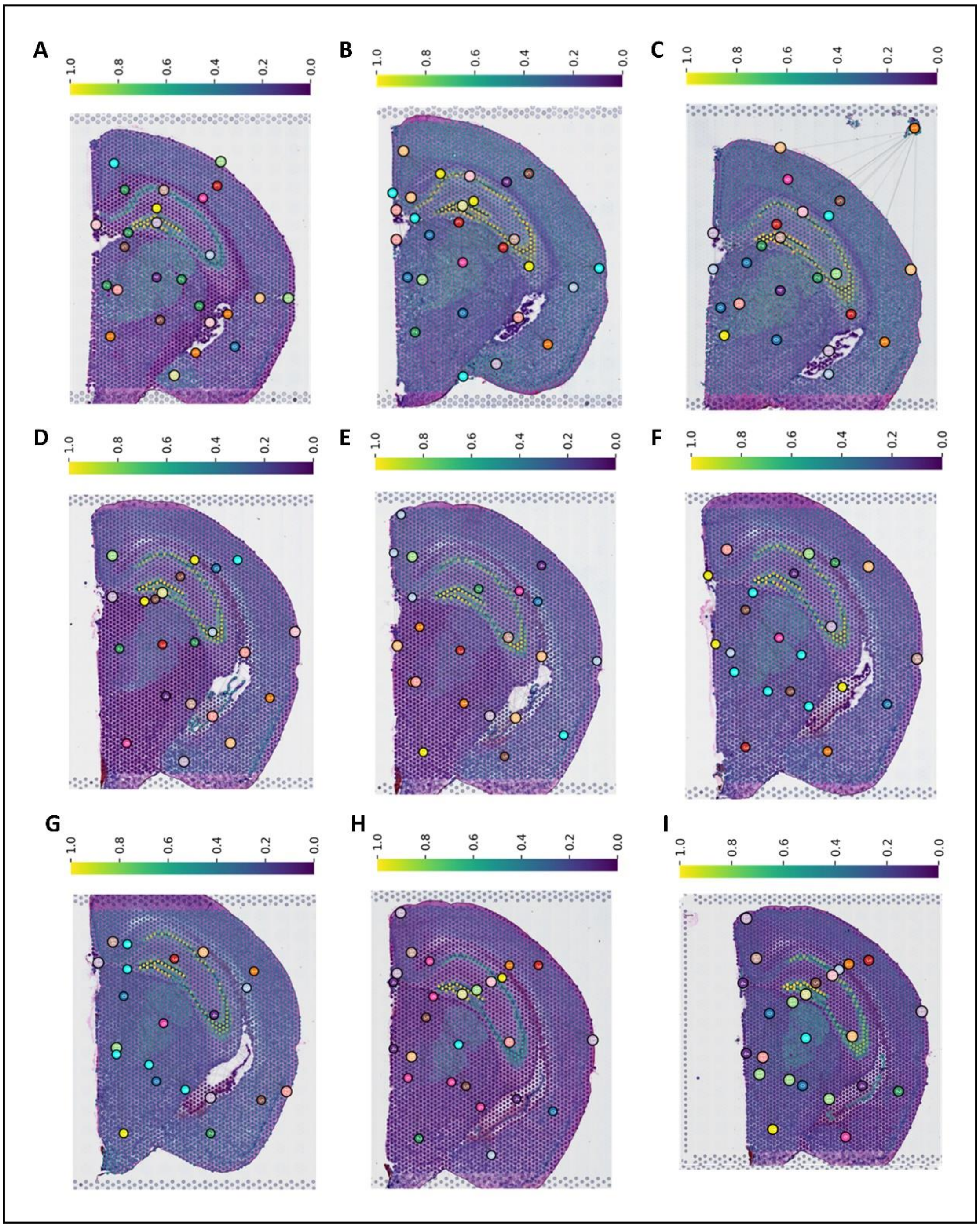

1.2 The planar diffusion pseudotime plots. **(A)** IVH_3d_3_20. **(B)** IVH_3d_3_50. **(C)** IVH_3d_3_110. **(D)** IVH_3d_3_130. **(E)** IVH_3d_3_150. **(F)** IVH_7d_2_50. **(G)** IVH_7d_2_80. **(H)** IVH_7d_2_110.

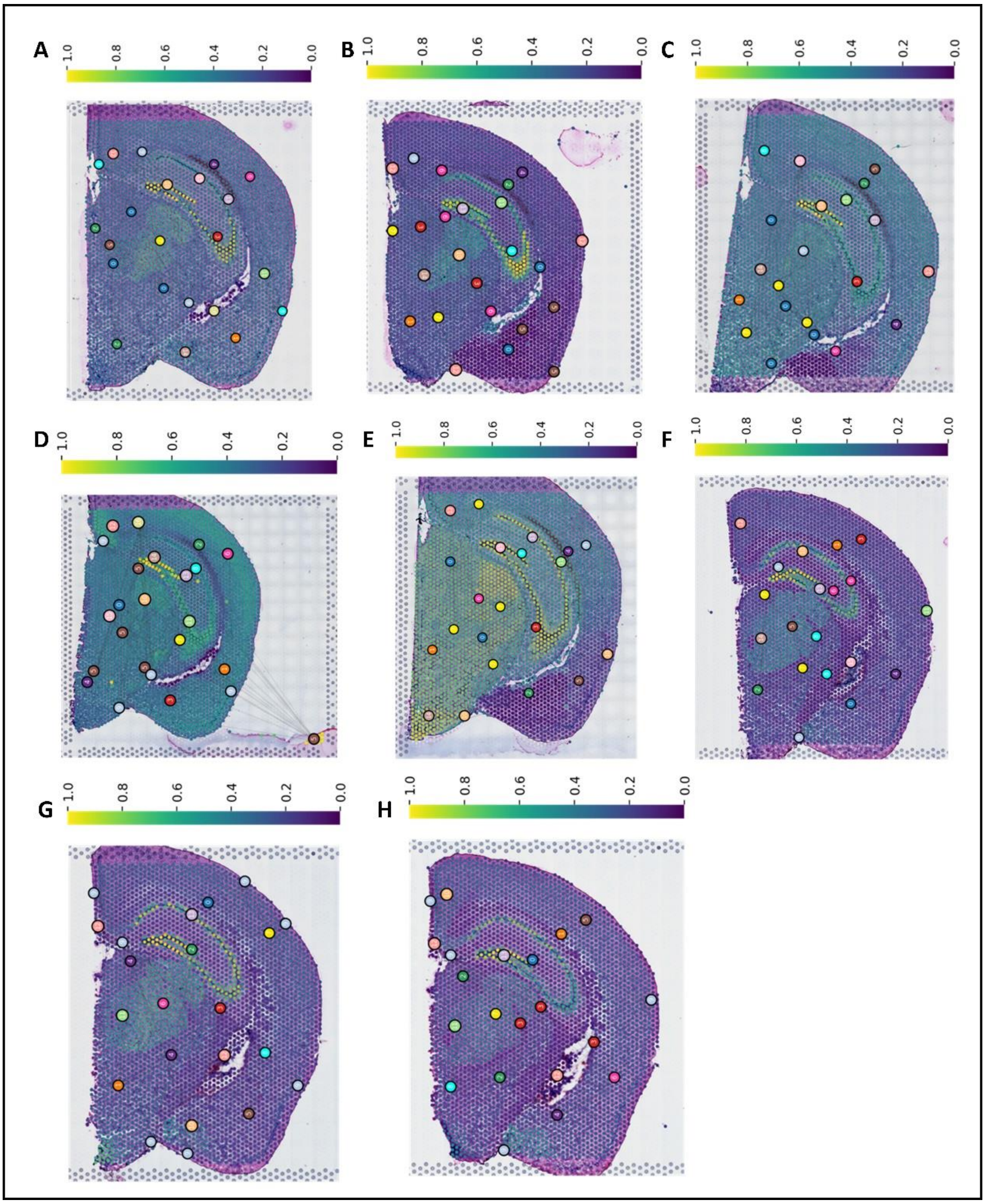

Figure 2 The planar global Pseudo-space-time trajectories

2.1 The planar global Pseudo-space-time trajectories. **(A)** IVH_S1_50. **(B)** IVH_S1_80. **(C)** IVH_S1_110. **(D)** IVH_1d_1_20. **(E)** IVH_1d_1_50. **(F)** IVH_1d_1_80.

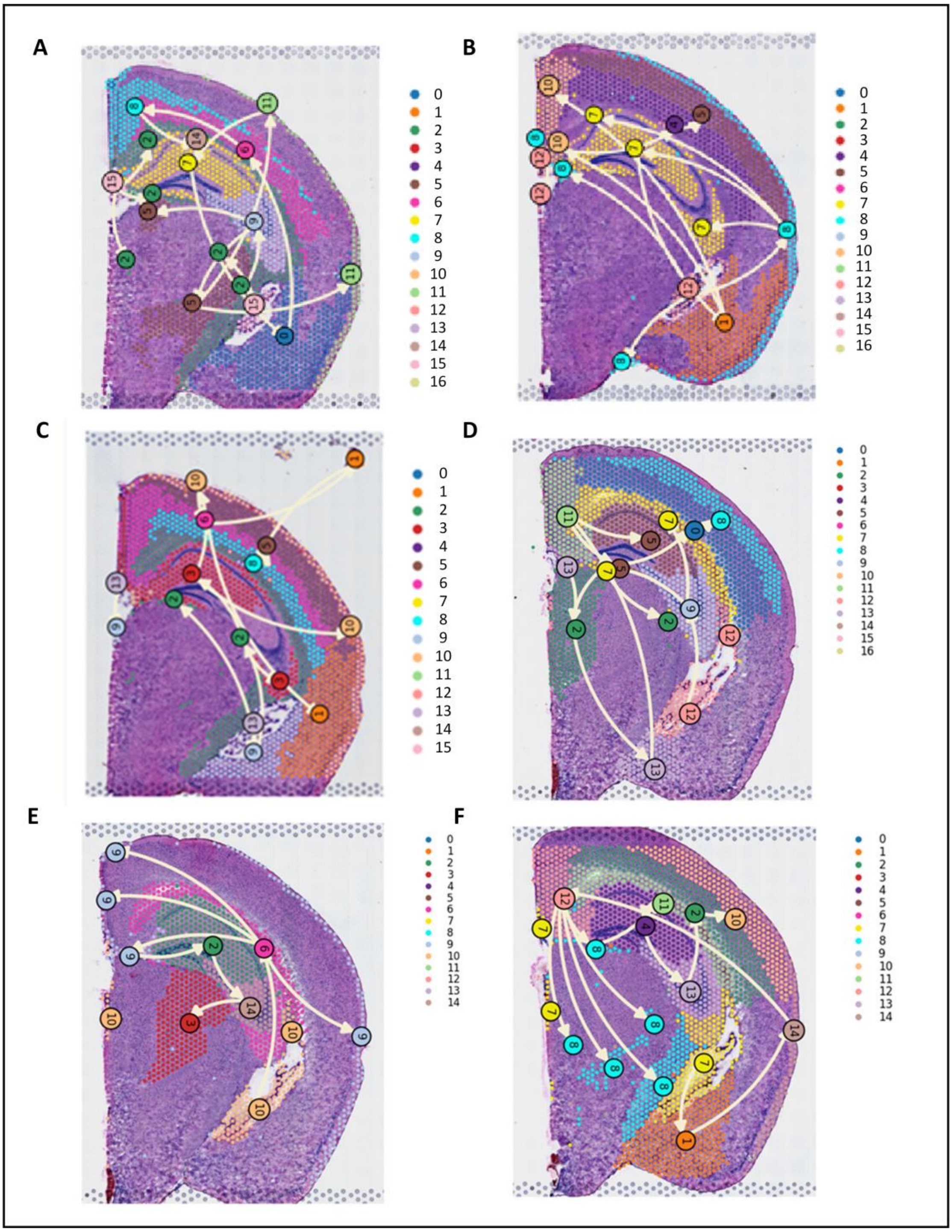

2.2 The planar global Pseudo-space-time trajectories. **(A)** IVH_1d_1_110. **(B)** IVH_1d_1_130. **(C)** IVH_1d_1_150. **(D)** IVH_3d_3_20. **(E)** IVH_3d_3_50. **(F)** IVH_3d_3_110.

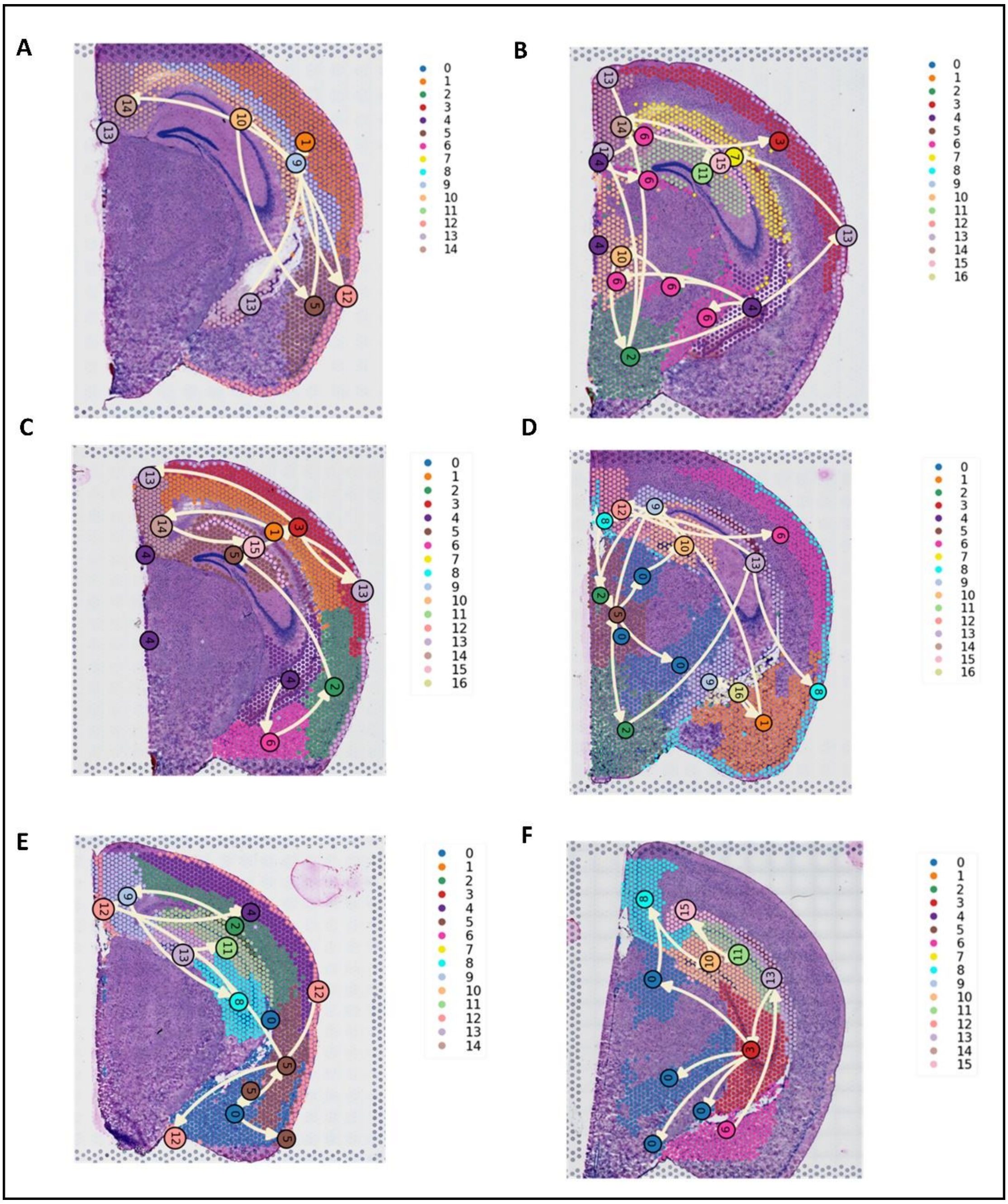

2.3 The planar global Pseudo-space-time trajectories. **(A)** IVH_3d_3_130. **(B)** IVH_3d_3_150. **(C)** IVH_7d_2_50. **(D)** IVH_7d_2_80. **(E)** IVH_7d_2_110.

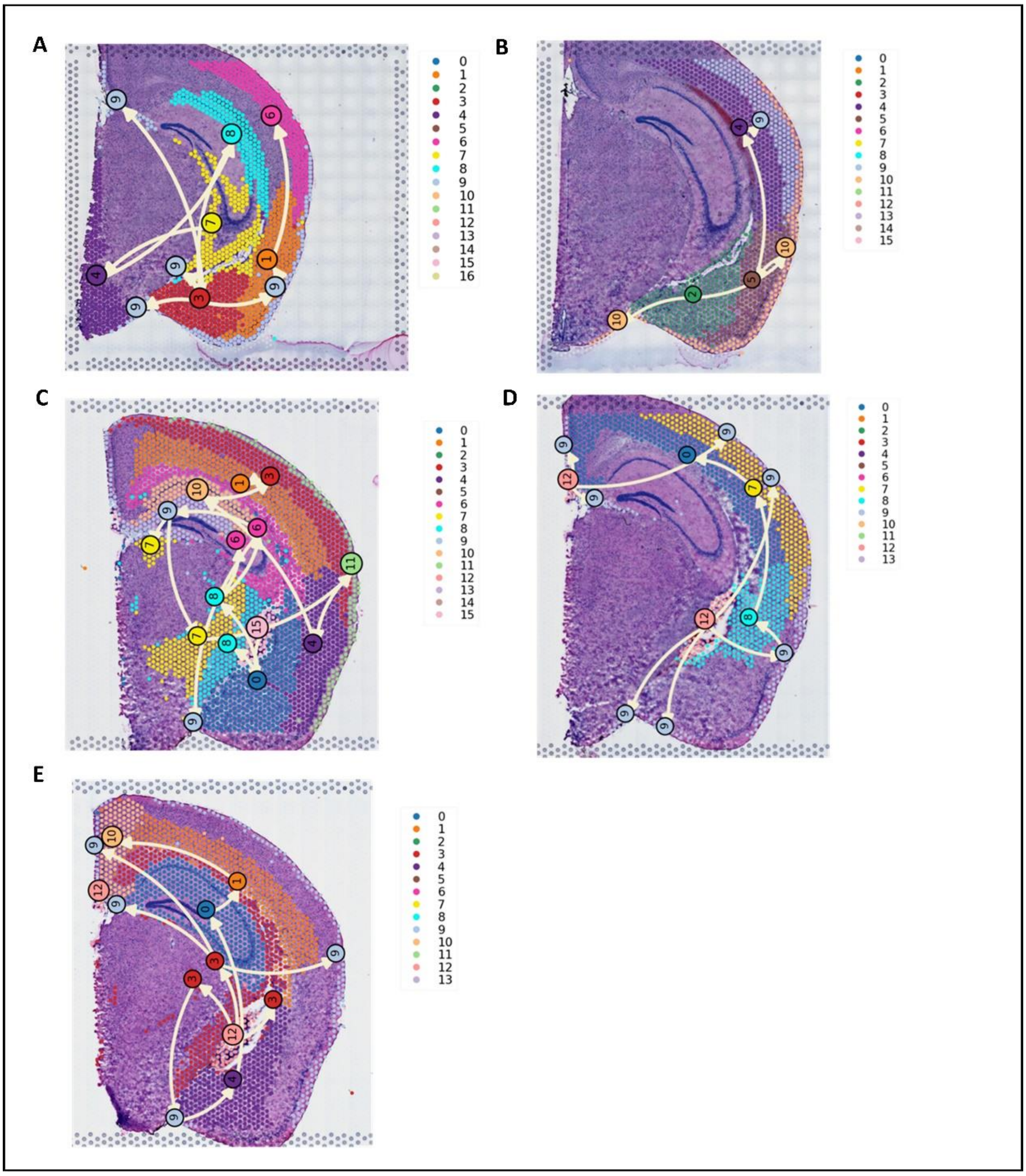

Figure 3 3D global Pseudo-space-time trajectories

3.1 3D global Pseudo-space-time trajectories. **(A)** IVH_S1. **(B)** Trajectory bundle I of IVH_1d. **(C)** Trajectory bundle II of IVH_1d.

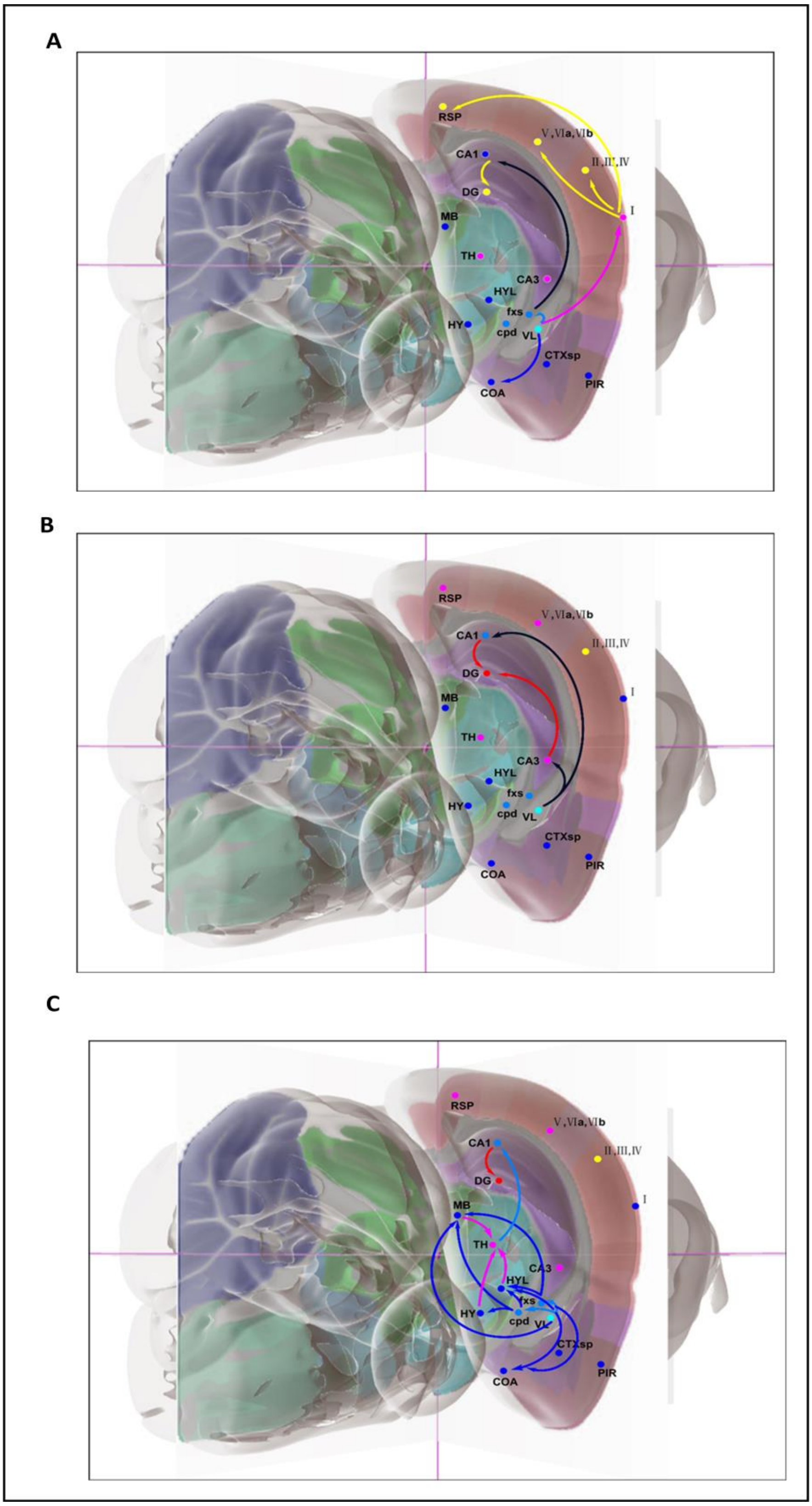

3.2 3D global Pseudo-space-time trajectories. **(A)** Trajectory bundle III of IVH_1d. **(B)** Trajectory bundle I of IVH_3d. **(C)** Trajectory bundle II of IVH_3d.

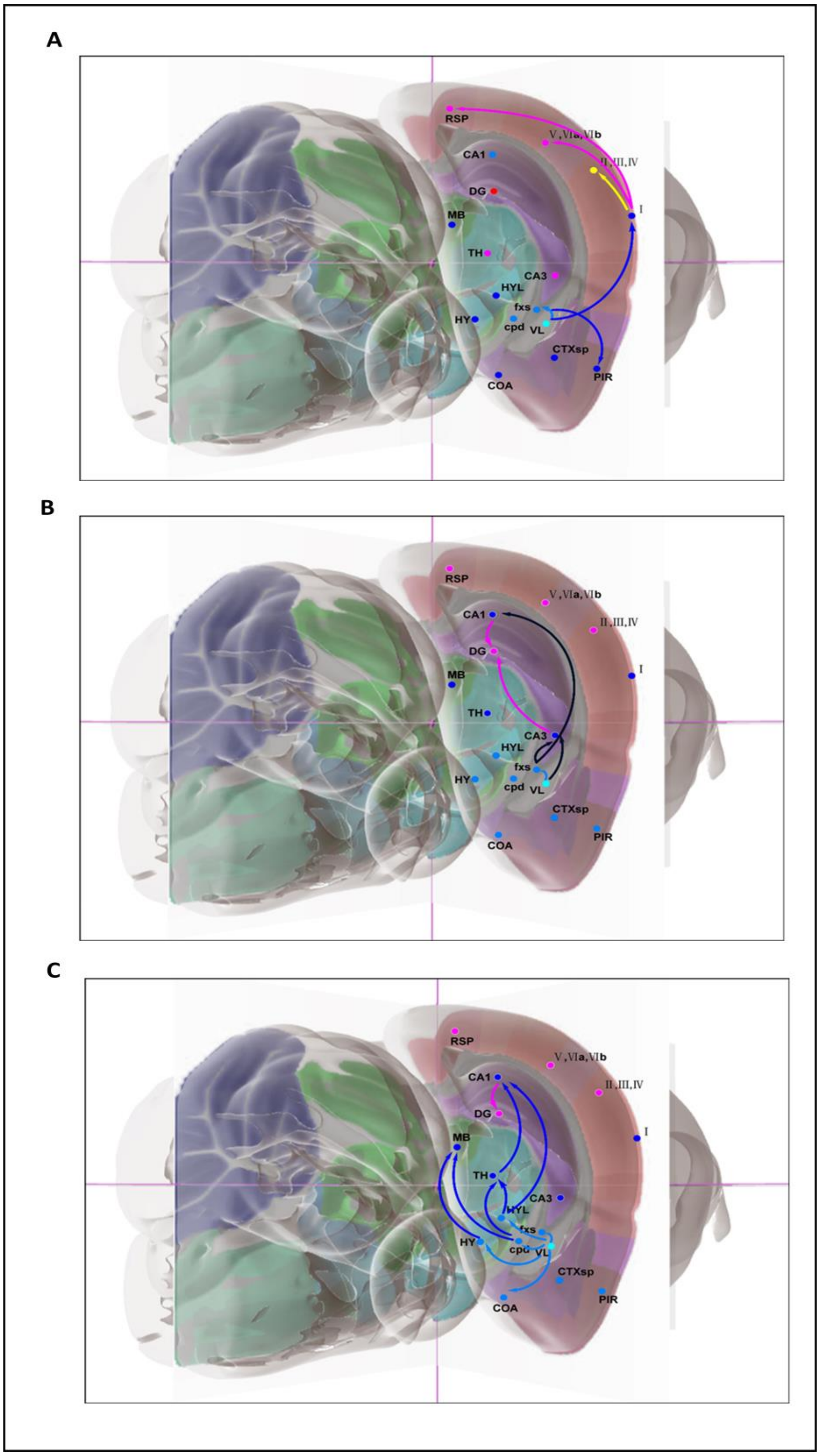

3.3 3D global Pseudo-space-time trajectories. **(A)** Trajectory bundle III of IVH_3d. **(B)** Trajectory bundle I of IVH_7d. **(C)** Trajectory bundle II of IVH_7d. **(D)** Trajectory bundle III of IVH_7d.

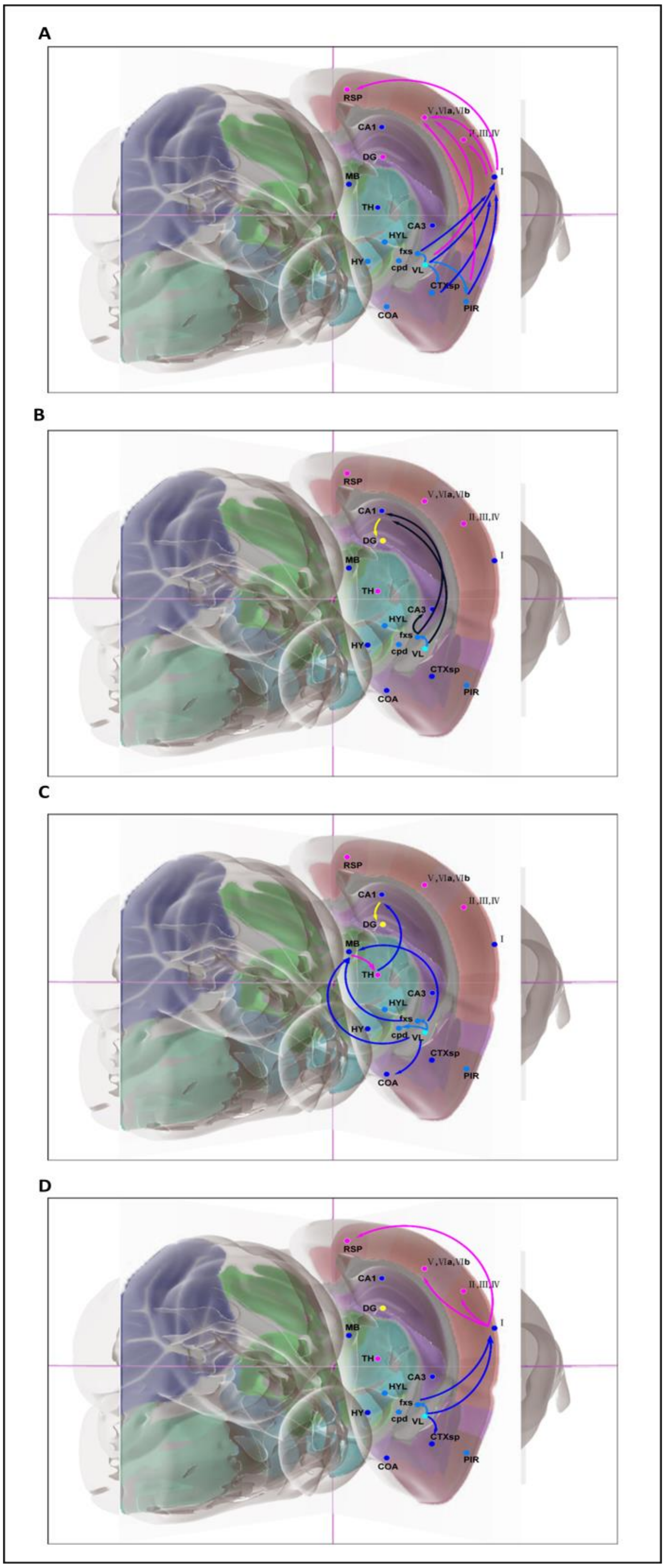

3. **Supplementary of Result 3 (SR3)**

Table 1 The planar upregulated and downregulated transition genes for each planner sub-trajectory at different time

https://github.com/JiayidaerBadai/Spatial-transcriptome.git

a. IVH_1d
b. IVH_3d
c. IVH_7d
d. IVH_S1

Table 2.1 The top 30 3D upregulated transition genes for each 3D sub-trajectory at different time

https://github.com/JiayidaerBadai/Spatial-transcriptome.git

a. IVH_1d
b. IVH_3d
c. IVH_7d
d. IVH_S1

Table 2.2 The top 30 3D downregulated transition genes for each 3D sub-trajectory at different time

https://github.com/JiayidaerBadai/Spatial-transcriptome.git

a. IVH_1d
b. IVH_3d
c. IVH_7d
d. IVH_S1

4. **Supplementary of Result 4 (SR4)**

Table 1 Cell subtypes corresponding the selected trajectory at different time

https://github.com/JiayidaerBadai/Spatial-transcriptome.git

Table 2 The marker gene sets of cell subtypes corresponding the selected trajectory at different time

https://github.com/JiayidaerBadai/Spatial-transcriptome.git

Table 3 The similarity between cell subtypes at different time on the same trajectory

https://github.com/JiayidaerBadai/Spatial-transcriptome.git

Table 4 The cell types that are as same as cell subtypes corresponding the selected trajectory at different time

https://github.com/JiayidaerBadai/Spatial-transcriptome.git

Table 5 The cell types shared by our identified cell subtypes at different time on the same trajectory.

https://github.com/JiayidaerBadai/Spatial-transcriptome.git

5. Supplementary of Result 5 (SR5)

Table 1 Upregulated/downregulated transition ligand-receptor sets

https://github.com/JiayidaerBadai/Spatial-transcriptome.git

1.1： IVH_1d upregulated transition ligand-receptor sets

1.2： IVH_1d downregulated transition ligand-receptor sets

1.3： IVH_3d upregulated transition ligand-receptor sets

1.4： IVH_3d downregulated transition ligand-receptor sets

1.5： IVH_7d upregulated transition ligand-receptor sets

1.6： IVH_7d downregulated transition ligand-receptor sets

1.7 ： IVH_S1 upregulated transition ligand-receptor sets

1.8 ： IVH_S1 downregulated transition ligand-receptor sets

Table 2 The interaction intensity value $Zscore$ (CCI) for the upregulated/downregulated transition ligand-receptor sets.

https://github.com/JiayidaerBadai/Spatial-transcriptome.git

2.1： The interaction intensity value $Zscore$ (CCI) for the IVH_1d upregulated transition ligand-receptor sets

2.2： The interaction intensity value $Zscore$ (CCI) for the IVH_1d downregulated transition ligand-receptor sets

2.3： The interaction intensity value $Zscore$ (CCI) for the IVH_3d upregulated transition ligand-receptor sets

2.4： The interaction intensity value $Zscore$ (CCI) for the IVH_3d downregulated transition ligand-receptor sets

2.5： The interaction intensity value $Zscore$ (CCI) for the IVH_7d upregulated transition ligand-receptor sets

2.6： The interaction intensity value $Zscore$ (CCI) for the IVH_7d downregulated transition ligand-receptor sets

2.7： The interaction intensity value $Zscore$ (CCI) for the IVH_S1 upregulated transition ligand-receptor sets

2.8： The interaction intensity value $Zscore$ (CCI) for the IVH_S1 downregulated

transition ligand-receptor sets

Table 3 The interaction value ZT converted from $Zscore$ (CCI) for the upregulated/downregulated transition ligand-receptor sets.
https://github.com/JiayidaerBadai/Spatial-transcriptome.git

3.1： The interaction intensity value ZT for the IVH_1d upregulated transition ligand-receptor sets

3.2： The interaction intensity value ZT for the IVH_1d downregulated transition ligand-receptor sets

3.3： The interaction intensity value ZT for the IVH_3d upregulated transition ligand-receptor sets

3.4： The interaction intensity value ZT for the IVH_3d downregulated transition ligand-receptor sets

3.5： The interaction intensity value ZT for the IVH_7d upregulated transition ligand-receptor sets

3.6： The interaction intensity value ZT for the IVH_7d downregulated transition ligand-receptor sets

3.7： The interaction intensity value ZT for the IVH_S1 upregulated transition ligand-receptor sets

3.8： The interaction intensity value ZT for the IVH_S1 downregulated transition ligand-receptor sets

Table 4 The $Density$ for each ligand-receptor of cell subtypes
https://github.com/JiayidaerBadai/Spatial-transcriptome.git

4.1： The average $Density$ for each ligand-receptor of IVH_1d upregulated transition ligand-receptor sets

4.2： The average $Density$ for each ligand-receptor of IVH_1d downregulated transition ligand-receptor sets

4.3： The average $Density$ for each ligand-receptor of IVH_3d upregulated transition ligand-receptor sets

4.4： The average $Density$ for each ligand-receptor of IVH_3d downregulated transition ligand-receptor sets

4.5： The average $Density$ for each ligand-receptor of IVH_7d upregulated transition ligand-receptor sets

4.6： The average $Density$ for each ligand-receptor of IVH_7d downregulated transition ligand-receptor sets

4.7： The average $Density$ for each ligand-receptor of IVH_S1 upregulated transition ligand-receptor sets

4.8： The average $Density$ for each ligand-receptor of IVH_S1 downregulated transition ligand-receptor sets

Table 5 The Kruskal-Wallis test for Density among these cell subtypes respectively on trajectory $\mathrm{VL} \rightarrow \mathrm{fxs} \rightarrow \mathrm{CA1} \rightarrow \mathrm{DG}$, $\mathrm{TH} \rightarrow \mathrm{CA1} \rightarrow \mathrm{DG}$ and $\mathrm{VL} \rightarrow \mathrm{I} \rightarrow (\mathrm{V}, \mathrm{VIa}, \mathrm{VIb})$.
https://github.com/JiayidaerBadai/Spatial-transcriptome.git

6. Supplementary of Result 6 (SR6)

Figure 1 The heatmap of mutual pathway sets for our identified cell subtypes

1.1 The heatmap of mutual pathway sets for trajectory VL→fxs→CA1→DG

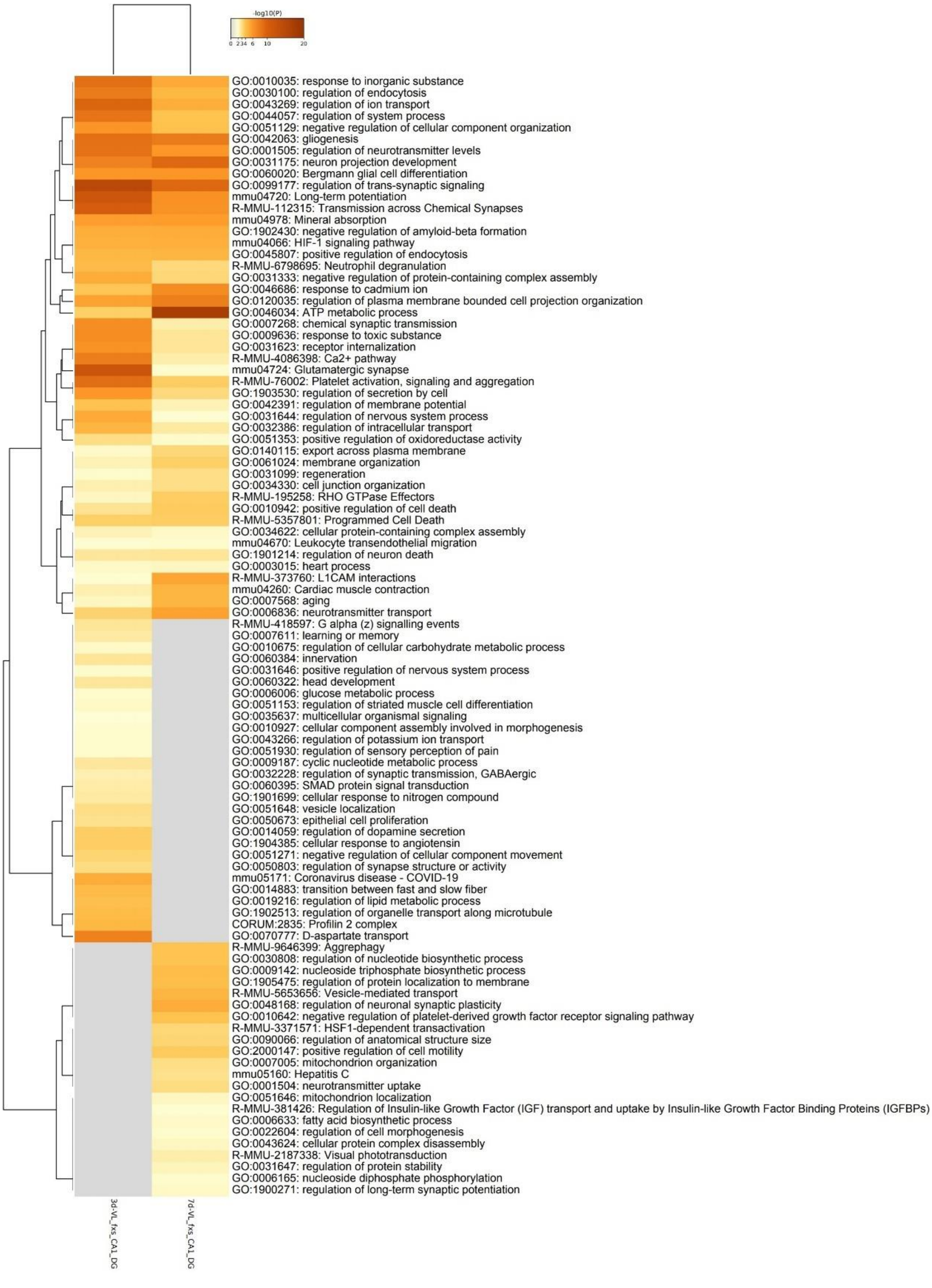
-log10(P)
0 2 3 4 6 10 20
GO:0010035: response to inorganic substance
GO:0030100: regulation of endocytosis
GO:0043269: regulation of ion transport
GO:0044057: regulation of system process
GO:0051129: negative regulation of cellular component organization
GO:0042063: gliogenesis
GO:0001505: regulation of neurotransmitter levels
GO:0031175: neuron projection development
GO:0060020: Bergmann glial cell differentiation
GO:0099177: regulation of trans-synaptic signaling
mmu04720: Long-term potentiation
R-MMU-112315: Transmission across Chemical Synapses
mmu04978: Mineral absorption
GO:1902430: negative regulation of amyloid-beta formation
mmu04066: HIF-1 signaling pathway
GO:0045807: positive regulation of endocytosis
R-MMU-6798695: Neutrophil degranulation
GO:0031333: negative regulation of protein-containing complex assembly
GO:0046686: response to cadmium ion
GO:0120035: regulation of plasma membrane bounded cell projection organization
GO:0046034: ATP metabolic process
GO:0007268: chemical synaptic transmission
GO:0009636: response to toxic substance
GO:0031623: receptor internalization
R-MMU-4086398: Ca2+ pathway
mmu04724: Glutamatergic synapse
R-MMU-76002: Platelet activation, signaling and aggregation
GO:1903530: regulation of secretion by cell
GO:0042391: regulation of membrane potential
GO:0031644: regulation of nervous system process
GO:0032386: regulation of intracellular transport
GO:0051353: positive regulation of oxidoreductase activity
GO:0140115: export across plasma membrane
GO:0061024: membrane organization
GO:0031099: regeneration
GO:0034330: cell junction organization
R-MMU-195258: RHO GTPase Effectors
GO:0010942: positive regulation of cell death
R-MMU-5357801: Programmed Cell Death
GO:0034622: cellular protein-containing complex assembly
mmu04670: Leukocyte transendothelial migration
GO:1901214: regulation of neuron death
GO:0003015: heart process
R-MMU-373760: L1CAM interactions
mmu04260: Cardiac muscle contraction
GO:0007568: aging
GO:0006836: neurotransmitter transport
R-MMU-418597: G alpha (z) signalling events
GO:0007611: learning or memory
GO:0010675: regulation of cellular carbohydrate metabolic process
GO:0060384: innervation
GO:0031646: positive regulation of nervous system process
GO:0060322: head development
GO:0006006: glucose metabolic process
GO:0051153: regulation of striated muscle cell differentiation
GO:0035637: multicellular organismal signaling
GO:0010927: cellular component assembly involved in morphogenesis
GO:0043266: regulation of potassium ion transport
GO:0051930: regulation of sensory perception of pain
GO:0009187: cyclic nucleotide metabolic process
GO:0032228: regulation of synaptic transmission, GABAergic
GO:0060395: SMAD protein signal transduction
GO:1901699: cellular response to nitrogen compound
GO:0051648: vesicle localization
GO:0050673: epithelial cell proliferation
GO:0014059: regulation of dopamine secretion
GO:1904385: cellular response to angiotensin
GO:0051271: negative regulation of cellular component movement
GO:0050803: regulation of synapse structure or activity
mmu05171: Coronavirus disease - COVID-19
GO:0014883: transition between fast and slow fiber
GO:0019216: regulation of lipid metabolic process
GO:1902513: regulation of organelle transport along microtubule
CORUM:2835: Profilin 2 complex
GO:0070777: D-aspartate transport
R-MMU-9646399: Aggrephagy
GO:0030808: regulation of nucleotide biosynthetic process
GO:0009142: nucleoside triphosphate biosynthetic process
GO:1905475: regulation of protein localization to membrane
R-MMU-5653656: Vesicle-mediated transport
GO:0048168: regulation of neuronal synaptic plasticity
GO:0010642: negative regulation of platelet-derived growth factor receptor signaling pathway
R-MMU-3371571: HSF1-dependent transactivation
GO:0090066: regulation of anatomical structure size
GO:2000147: positive regulation of cell motility
GO:0007005: mitochondrion organization
mmu05160: Hepatitis C
GO:0001504: neurotransmitter uptake
GO:0051646: mitochondrion localization
R-MMU-381426: Regulation of Insulin-like Growth Factor (IGF) transport and uptake by Insulin-like Growth Factor Binding Proteins (IGFBPs)
GO:0006633: fatty acid biosynthetic process
GO:0022604: regulation of cell morphogenesis
GO:0043624: cellular protein complex disassembly
R-MMU-2187338: Visual phototransduction
GO:0031647: regulation of protein stability
GO:0006165: nucleoside diphosphate phosphorylation
GO:1900271: regulation of long-term synaptic potentiation
3d-VL_fxs_CA1_DG
7d-VL_fxs_CA1_DG

1.2 The heatmap of mutual pathway sets for trajectory TH→CA1→DG

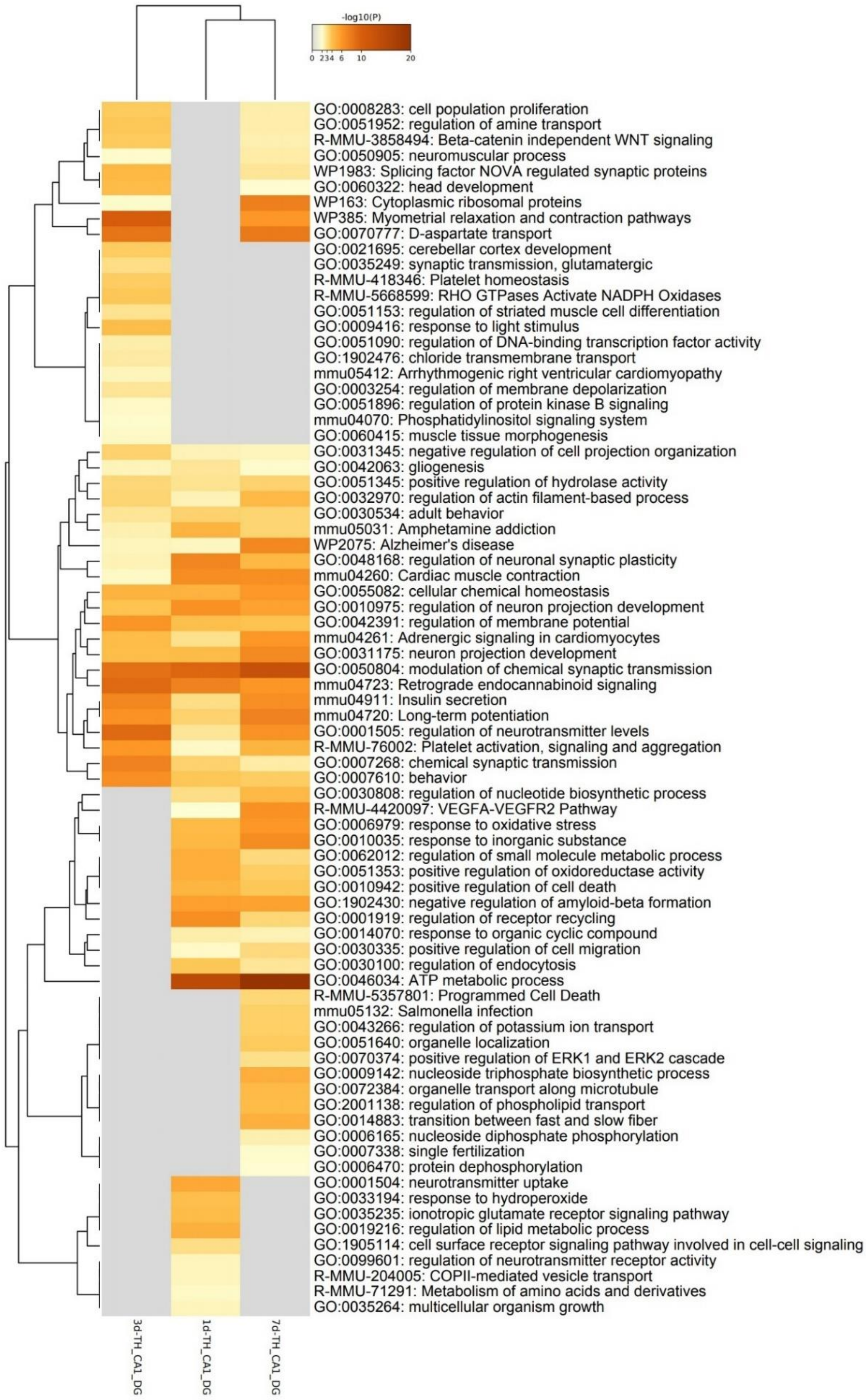

1.3 The heatmap of mutual pathway sets for trajectory VL→I→(V,VIa,VIb)

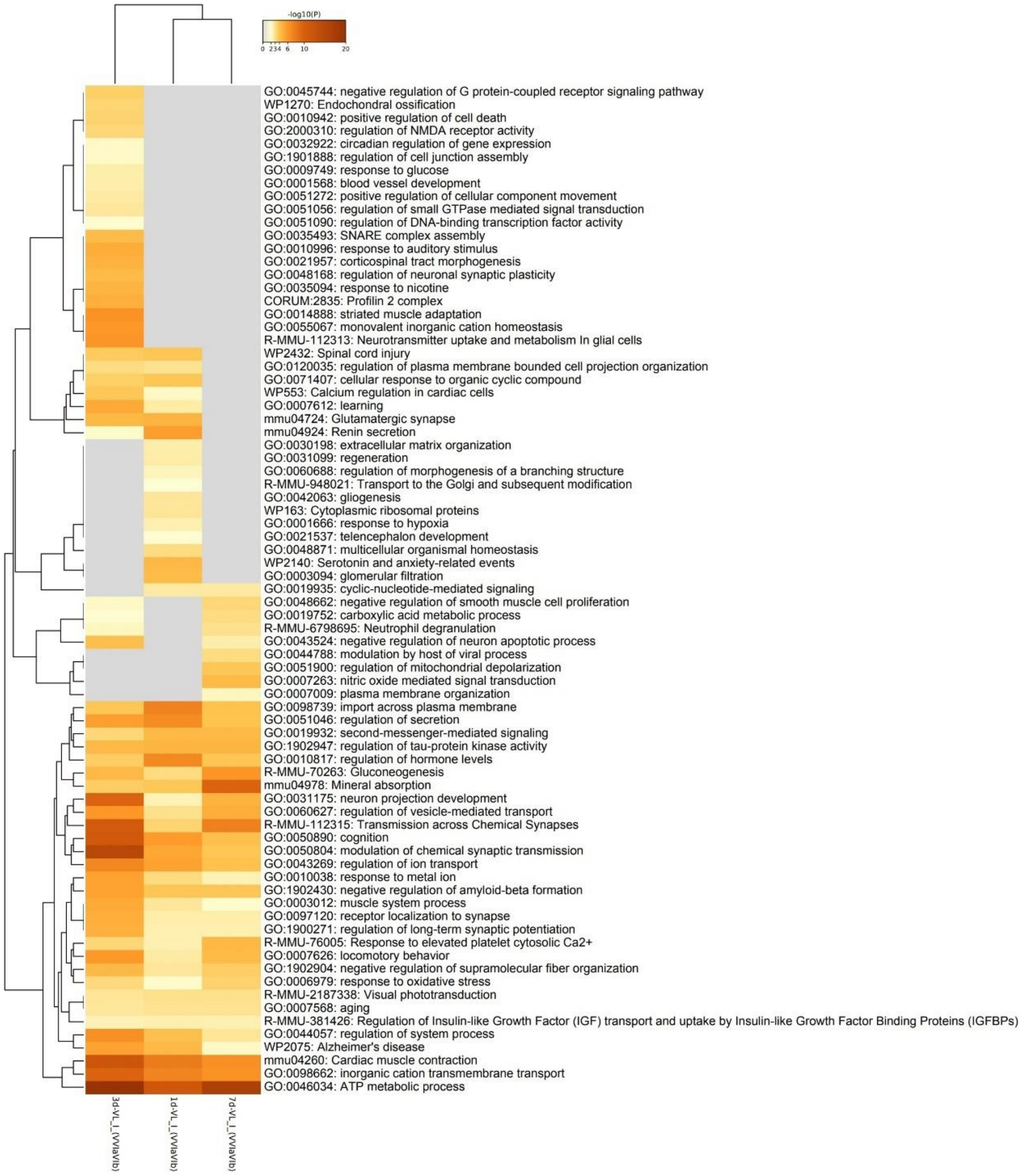

Figure 2 The heatmap of mutual pathway sets for similar cell types.

2.1 The heatmap of mutual pathway sets for similar cell types of trajectory VL→fxs→CA1→DG

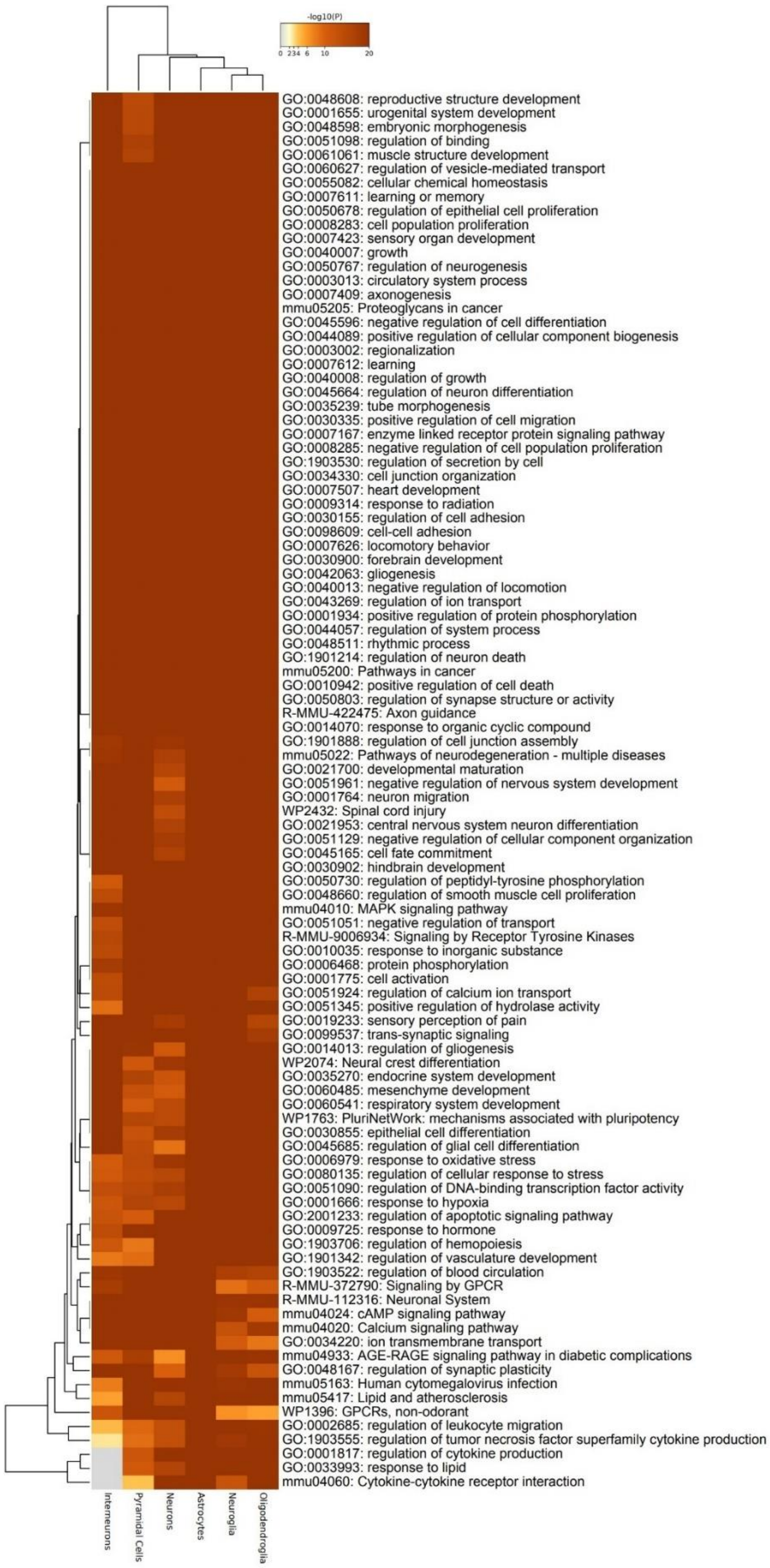

2.2 The heatmap of mutual pathway sets for similar cell types of trajectory TH→CA1→DG

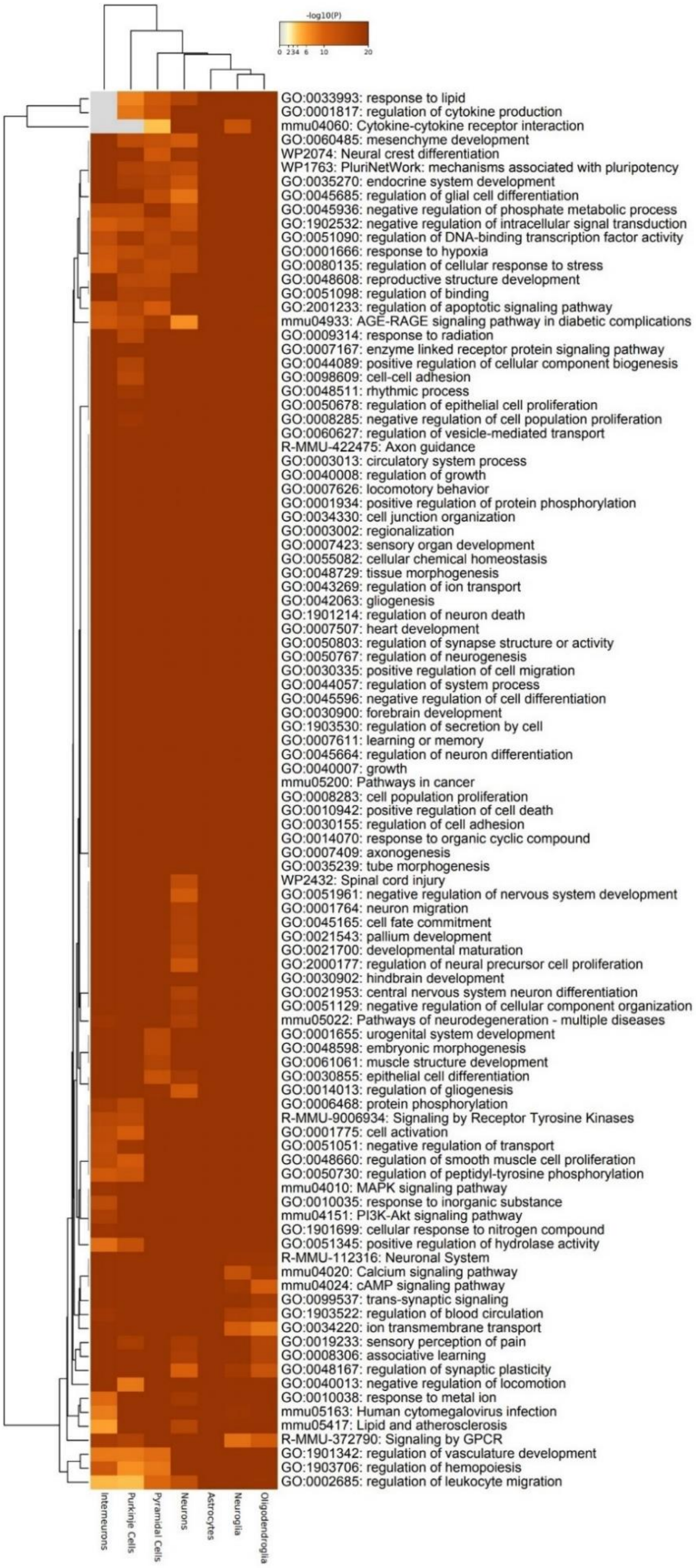

2.3 The heatmap of mutual pathway sets for similar cell types of trajectory VL→I→(V,VIa,VIb)

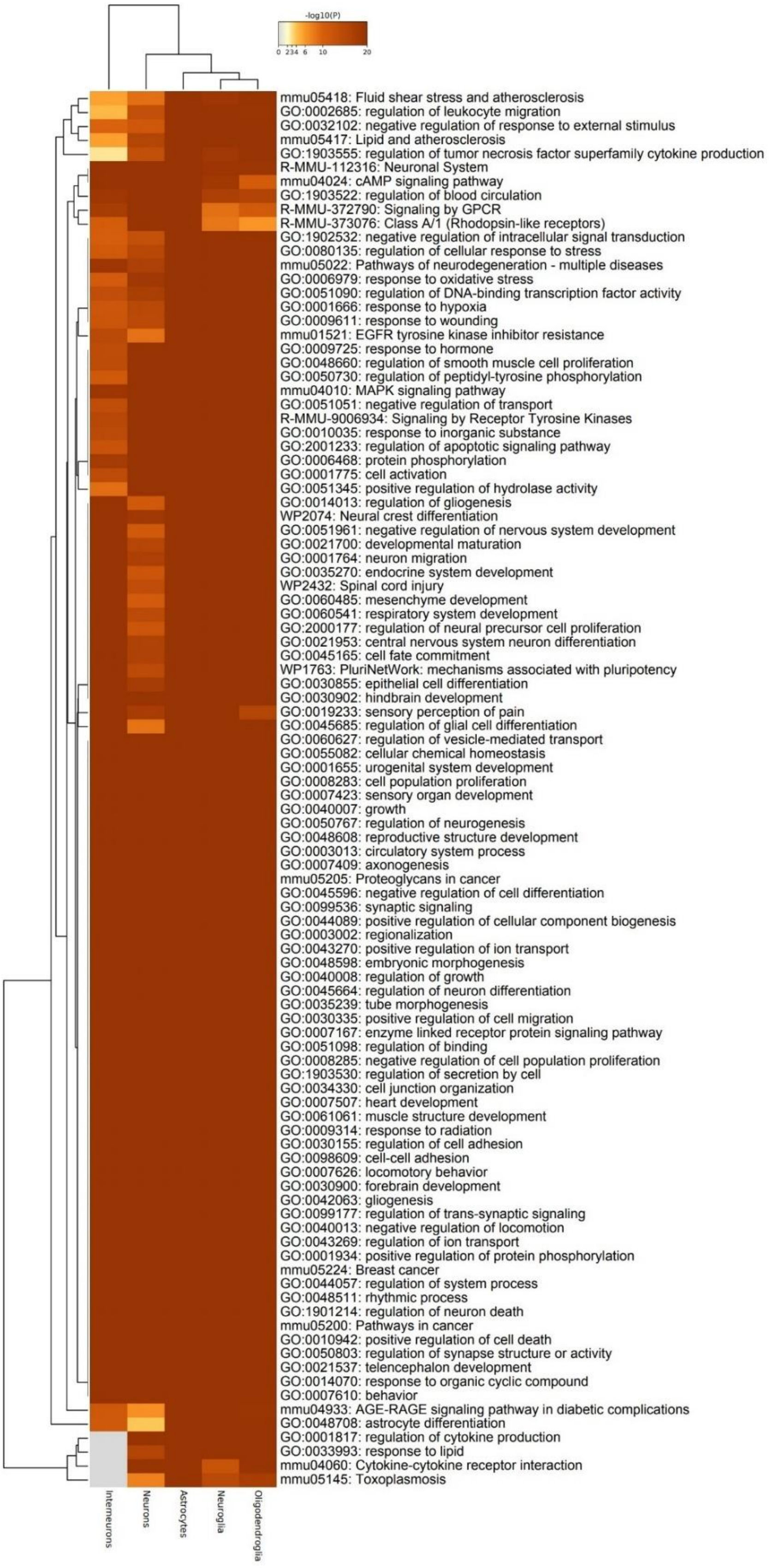

Table 1 Marker genes of similar cell types for our identified cell subtypes
https://github.com/JiayidaerBadai/Spatial-transcriptome.git

Table 2 The mutual pathway sets for our identified cell subtypes
https://github.com/JiayidaerBadai/Spatial-transcriptome.git

2.1 The mutual pathway sets for trajectory VL→fxs→CA1→DG

2.2 The mutual pathway sets for trajectory TH→CA1→DG

2.3 The mutual pathway sets for trajectory VL→I→(V,VIa,VIb)

Table 3 The mutual pathway sets for similar cell types
https://github.com/JiayidaerBadai/Spatial-transcriptome.git

3.1 The mutual pathway sets for similar cell types of trajectory VL→fxs→CA1→DG

3.2 The mutual pathway sets for similar cell types of trajectory TH→CA1→DG

3.3 The mutual pathway sets for similar cell types of trajectory VL→I→(V,VIa,VIb)

Table 4 The mutual pathway set between our identified cell subtypes and similar cell types https://github.com/JiayidaerBadai/Spatial-transcriptome.git

II. Supplementary of Methods (SM)

This supplementary of Methods section consists of 11 supplementary files, which is listed as below.

Figure1 IVH model

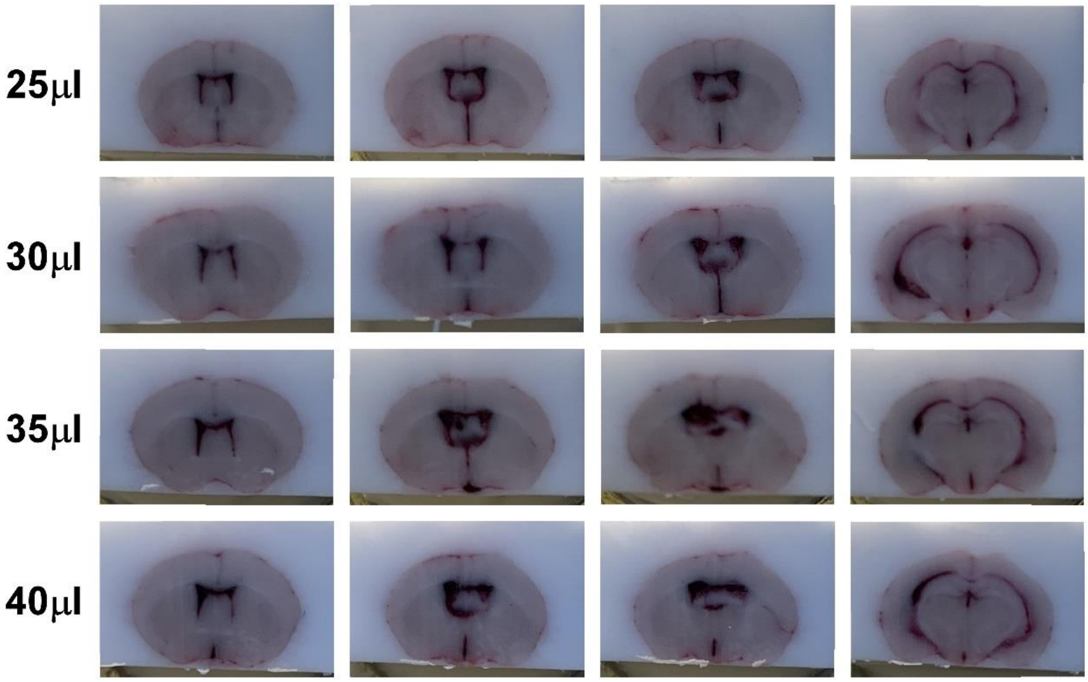

Table 1 IVH sample grouping, library patching strategy and permeabilization time（min）

group	sample	Permeabilization time（min）
1. IVH_1d	IVH_1d_1_20	25.5min
	IVH_1d_1_50	25.5min
	IVH_1d_1_80	25.5min
	IVH_1d_1_110	25.5min
	IVH_1d_1_130	25.5min
	IVH_1d_1_150	25.5min
2. IVH_3d	IVH_3d_3_20	19min
	IVH_3d_3_50	19min
	IVH_3d_3_110	19min
	IVH_3d_3_130	19min
	IVH_3d_3_150	19min
3. IVH_7d	IVH_7d_2_50	25min
	IVH_7d_2_80	25min
	IVH_7d_2_110	25min
4. IVH_C1	IVH_C1_50	25min
	IVH_C1_110	25min
5. IVH_S1	IVH_S1_50	25min
	IVH_S1_80	25min
	IVH_S1_110	25min

Table 2 Parameter Definition for 3D global Pseudo-space-time trajectory reconstruction algorithm

No.	Definition	Description
1.	$Time_t\ , t \in [1,5]$	Time t after IVH
2.	$slide_n\ , n \in [1, N]$	Slide n.
3.	$Trajectory^n \equiv G^n = (V^n, E^n),\ n \in [1, N]$	The set of planar pseudo-space-time trajectories on the slide n, equivalent to a Directed Acyclic Graph G^n on the slide n which is composed of nodes V^n and directed edges E^n.
4.	$V^n(v_i^n \in V^n, i \in [1, N])$	Nodes V^n on the graph G^n represents brain regions on the slide n, v_i^n represents brain region I on the slide n.
5.	$E^n(e_{ij}^n \in E^n, i \in [1, N], \mathrm{j} \in [1, N])$	Directed edges E^n on the graph G^n represents the set of directed edge e_{ij}^n from the brain region I to the brain region j on the slide n.
6.	$DPT_{v_i^n}^m,\ n \in [1, N],\ m \in [1, N],\ i \in [1, N],\ DPT_{v_i^n}^m \in [0,1]$	$DPT_{v_i^n}^m$ represents the diffusion pseudotime of each spot in node (brain region) *i.*
7.	$Avg\ \ DPT_{v_i^n} = \frac{\sum_{m=1}^{N} DPT_{v_i^n}^m}{m}$ (1)	The average diffusion pseudotime for each planar brain region.
8.	$3D\_dpt_{v_i} = \frac{\sum_{n=1}^{N} Avg\ \ DPT_{v_i^n}}{N}$ (2)	3D diffusion pseudotime of brain region *i.*
9.	imagerow ($x_{v_i^n}^m$), imagecol ($y_{v_i^n}^m$)	Planar coordinates of each spot in node (brain region) *i.*

10.	$z_{v_i^n}=\begin{cases} n=1\ (Time_t\_20),\ 1 \\ n=2\ (Time_t\_50),\ 2 \\ n=3\ (Time_t\_80),\ 3 \\ n=4\ (Time_t\_110),\ 4 \\ n=5\ (Time_t\_130),\ 5 \\ n=6(Time_t\_150),\ 6 \end{cases}$	Height coordinates of node (brain region) *I* of each slide.
11.	$(Avg\_x^m_{v_i^n}, Avg\_y^m_{v_i^n}) = (\frac{(x^1_{v_i^n}+x^2_{v_i^n}+\cdots+x^m_{v_i^n})}{m}, \frac{(y^1_{v_i^n}+y^2_{v_i^n}+\cdots+y^m_{v_i^n})}{m})$ (3)	Planar Centroid coordinates of node v_i on the slide n with m spots.
12.	v_1^n	v_1^n represents the starting node (brain region) of the set of planar pseudo-space-time trajectories on the slide n.
13.	$(x_i, y_i, z_i) = (\frac{(x^m_{v_i^1}+x^m_{v_i^2}+\cdots+x^m_{v_i^n})}{n}, \frac{(y^m_{v_i^1}+y^m_{v_i^2}+\cdots+y^m_{v_i^n})}{n})$ (4)	3D Centroid coordinates of v_i
14.	$v_f^n,\ f \in F^n$	v_f^n represents ending node (brain region) of planar pseudo-space-time trajectory on the slide n. F^n represents ending node brain region set of planar pseudo-space-time trajectories.
15.	$v_i = (l_i, p_i),\ l_i \in [1,5],\ p_i \in [1,3]$	$l_i \in [1,5]$ represents that the node (brain region) v_i is divided into 5 levels according to the average diffusion pseudotime of brain region. $p_i \in [1,3]$ represents that according to the trajectory (path) from the starting node 1 to the ending node f, node v_i on trajectory $T^n_{1-..-i-..-f} \in Trajectory^n$ is divided into 3 categories. p_1 contains

			"fxs", "CA3", "CA1", "DG". p_2 contains "COA", "HY", "cpd", "fxs", "HYL", "TH", "MB", "CA1", "CA3", "DG". p_3 contains "CTXsp", "PIR", "I", "II_III_IV", "V_VIa_VIb", "RSP".
16.	$Trajectory^{common}$		A common set of 3D pseudo-space-time trajectories.
17.	$B^n,\ n \in [1, N]$		The starting nodes of the set of planar pseudo-space-time trajectories $Trajectory^n$.
18.	$C^n,\ n \in [1, N]$		Descendant node set.
19.	$H^n,\ n \in [1, N]$		Candidate descendant node set.
20.	D^n		Final descendant node set.
21.	$dis_{ij} = \sqrt{(x_i - x_j)^2 + (y_i - y_j)^2 + (z_i - z_j)^2}$	(5)	Distance between node I and node j, $i \in [1, N]$, j$\in [1, N]$
22.	Dis_i		Distance set of node i.

Table 3 3D global Pseudo-space-time trajectory reconstruction algorithm

Algorithm 3D Global Pseudo-space-time Trajectory Reconstruction Algorithm

Input: $Trajectory^n$, v_i^n, e_i^n, p_i, $DPT_{v_i^n}^m$, imagerow ($x_{v_i^n}^m$), imagecol ($y_{v_i^n}^m$)

For each pseudo-space-time trajectories index n:

  For each brain region *I* in pseudo-space-time trajectories:

    Calculate the the average diffusion pseudotime $Avg\ DPT_{v_i^n}$ by Eq.1

For each brain region *I* in pseudo-space-time trajectories:

  Calculate the 3D diffusion pseudotime $3D\_dpt_{v_i}$ by Eq.2

Divide the $3D\_dpt_i$ by step size (0.0889104 for brain regions in p_3 and 0.1462136 for brain regions in p_1 & p_2) to obtain the diffusion pseudotime level l_i for each 3D brain region from 0 to 6 level.

Insert v_1 into set $Trajectory^{common}$

For each pseudo-space-time trajectories index *n* in p_i:

  Insert v_1 into set B^n

For *length* from *1* to *max_length*:

  $Min\_length = \{\infty\}^N$, $min\_index = \{0\}^N, C = \{\emptyset\}^N, H = \{\emptyset\}^N, D = \{\emptyset\}^N$

  For each pseudo-space-time trajectories index *n*:

    For each vector *I* in B^n:

      For each vector *j* in e_i^n:

        Insert vector *j* into set C^n

        If $l_i = length + n$:

          Insert vector *j* into set H^n

          If $v_i ==$ "TH" , $v_j ==$ "CA1" or $v_j ==$ "CA3"

            Insert vector *j* into set D^n

            Calculate the 3D Centroid coordinates of v_i by Eq.3-4, and distance dis_{ij}^n between vector *I* and vector *j* by Eq.5

            If $dis_{ij}^n < Min\_length$:

              $Min\_length^n = dis_{ij}^n$, $min\_index^n = j$

  If $\cup_{k=1}^N D^k = \emptyset$:

$$D = min\_index$$

  If $\cup_{k=1}^N C^k = \emptyset$:

    Break

  Else if $\cup_{k=1}^N H^k = \emptyset$:

    Help

    Else:

      For each pseudo-space-time trajectories index *n*:

        For each vector *I* in B^n:

          For each vector *j* in D^n:

            Insert edge e_{ij} into $Trajectory^{common}$

        $B^n = D^n$

36: **Output**: $Trajectory^{common}$

Table 4 Parameter Definition for the algorithm to identify a cell subtype and Similarity algorithm for cell subtypes

No.	Definition		Description
1.	$CellT_{\mathrm{Trajectory}_i}^{time_n}$		A cell subtype on $\mathrm{Trajectory}_i$ at different time, time represents the day (Sham, 1d, 3d and 7d). (SR4.Table 1)
2.	$GeneS_{\mathrm{Trajectory}_i}^{time_n} = (GeneNames, Symbols)$		A set of marker genes corresponding to $CellT_{\mathrm{Trajectory}_i}^{time_n}$. (SR4.Table 2)
3.	$Gp_{\mathrm{Trajectory}_i}^{time_n}$		The top 30 upregulated transition genes of 3D sub-trajectory $\mathrm{Trajectory}_i$ at $time_n$. (SR3. Table 2.1)
4.	$Gn_{\mathrm{Trajectory}_i}^{time_n}$		The top 30 downregulated transition genes of 3D sub-trajectory $\mathrm{Trajectory}_i$ at $time_n$. (SR3. Table 2.2)
5.	$Gp_{\mathrm{a->b}}^{1d} \cup Gp_{\mathrm{b->c}}^{1d} = Gp_{\mathrm{a->b->c}}^{1d}$	(6)	The upregulated transition genes of 3D trajectory $\mathrm{a->b->c}$ at the first day.
6.	$Gn_{\mathrm{a->b}}^{1d} \cup Gn_{\mathrm{b->c}}^{1d} = Gn_{\mathrm{a->b->c}}^{1d}$	(7)	The downregulated transition genes of 3D trajectory $\mathrm{a->b->c}$ at the first day.
7.	$Gp_{\mathrm{a->b->c}}^{1d} \cup Gn_{\mathrm{a->b->c}}^{1d} = G_{\mathrm{a->b->c}}^{1d} = \{g1+, g2-, g3+, \ldots g\mathrm{i}+\}$	(8)	$G_{\mathrm{a->b->c}}^{1d}$ describes a cell subtype in the trajectory (a->b->c) at the first day. $g\mathrm{i}$ Represents the gene, sign describes upregulation or downregulation.
8.	$GeneNames = \{g_0, g_1, g_2 \ldots g_j\}$	(9)	Represents the set of gene names, the element of the set is

			represented by g_j.
9.	$Symbols = \{s_0, s_1, s_2 \ldots s_j\}$	(10)	Represents the set of symbols, the element of the set is represented by s_j. The positive and negative of s_j represents whether it is positively or negatively correlated with the key trajectory.
10.	$Similarity_{\text{Trajectory}_i}^{time_{mn}} = \left(\frac{GeneS_{\text{Trajectory}_i}^{time_m} \cdot GeneS_{\text{Trajectory}_i}^{time_n}}{\left\lvert GeneS_{\text{Trajectory}_i}^{time_m} \cup GeneS_{\text{Trajectory}_i}^{time_n}\right\rvert} + 1\right)/2$	(11)	Eq.11-13 calculate the similarity between different cell subtypes $CellT_{\text{Trajectory}_i}^{time_n}$. (Figure 4 and SR4.Table 3)
	$GeneS_{\text{Trajectory}_i}^{time_m} \cdot GeneS_{\text{Trajectory}_i}^{time_n} = \sum(g_{j_m}, s_{j_m}) \cdot (g_{j_n}, s_{j_n})$	(12)	
	$(g_{j_m}, s_{j_m}) \cdot (g_{j_n}, s_{j_n}) = \begin{cases} -1, & (s_{j_m} != s_{j_n})\&\&(g_{j_m} = g_{j_n}) \\ 0, & (g_{j_m} \notin (g_{j_m} \cap g_{j_n})\&\&(g_{j_n} \notin (g_{j_m} \cap g_{j_n}) \\ 1, & (s_{j_m} = s_{j_n})\&\&(g_{j_m} = g_{j_n}) \end{cases}$	(13)	

Table 5 The algorithm to identify a cell subtype

**Algorithm** The algorithm to identify a cell subtype
**Input:** Trajectory_i (Trajectory a->b->c), $Gp_{\text{Trajectory}_i}^{time_n}$, $Gn_{\text{Trajectory}_i}^{time_n}$
**For** each sub trajectory $(\text{a}{-}{>}\ \text{b}, \text{b}{-}{>}\ \text{c})$ in Trajectory_i , $index\ n$:
Calculate the upregulated transition genes $Gp_{\text{Trajectory}_i}^{time_n}$ $(Gp_{\text{a}->\text{b}->\text{c}}^{1d})$ by Eq.6
Calculate the downregulated transition genes $Gn_{\text{Trajectory}_i}^{time_n}$ $(Gn_{\text{a}->\text{b}->\text{c}}^{1d})$ by Eq.7
**For** $Gp_{\text{Trajectory}_i}^{time_n}$ $(Gp_{\text{a}->\text{b}->\text{c}}^{1d})$ and $Gn_{\text{Trajectory}_i}^{time_n}$ $(Gn_{\text{a}->\text{b}->\text{c}}^{1d})$:
Calculate the union of these two sets $GeneS_{\text{Trajectory}_i}^{time_n}$ $(G_{\text{a}->\text{b}->\text{c}}^{1d})$ by Eq.8, if different plane slices had the same gene with a different sign (upregulated or downregulated), we chose the gene whose absolute value was the greatest
**Output**: $GeneS_{\text{Trajectory}_i}^{time_n}$

Table 6 Similarity algorithm for cell subtypes

Algorithm Similarity algorithm for cell subtypes

Input: $GeneS_{\mathrm{Trajectory}_i}^{time_n}$, $GeneNames = \{g_0, g_1, g_2 \dots g_j\}, Symbols = \{s_0, s_1, s_2 \dots s_j\}$ Eq.9-10

For each $GeneS_{\mathrm{Trajectory}_i}^{time_n}, index\ i, n$:

Calculate the similarity $Similarity_{\mathrm{Trajectory}_i}^{time_{m\_n}}$ between cell subtypes $CellT_{\mathrm{Trajectory}_i}^{time_n}$ at different time on the same trajectory by Eq.11-13

Output: $Similarity_{\mathrm{Trajectory}_i}^{time_{m\_n}}$

Table 7 Parameter Definition for Cell-cell communication strength (Density) algorithm

No.	Definition		Description
1.	LR_n		Ligand-receptor n. (SR5 Table 1.1 – 1.8)
2.	$Zscore_i^{LR_n}, i \in (1, N)$		Represents the interaction intensity of ligand-receptor (LR_n) at each point i on the section. (SR5 Table 2.1 - 2.8)
3.	$ZT_i^{LR_n}$		Represents the discrete interaction intensity after conversion of the continuous interaction intensity $Zscore_i^{LR_n}$. (SR5 Table 3.1-3.8)
4.	$ZTTotal$		Represents the sum of the interaction intensity $ZT_i^{LR_n}$ of N points in the corresponding position. (SR5 Table 3.1-3.8)
5.	A		Represents the number of points for each brain region pair.
6.	B		Represents the number of points for all brain regions.
7.	$Density^{LR_n}$		Represents the density of the interaction intensity of the ligand-receptor (LR_n) of cell type over the interaction intensity of all brain regions. (SR5 Table 4.1-4.8)
8.	$ZT_i^{LR_n} = \left\{ \begin{array}{ll} Zscore_i^{LR_n} < -3 & 0 \\ -3 \le Zscore_i^{LR_n} < -2 & 1 \\ -2 \le Zscore_i^{LR_n} < -1 & 2 \\ -1 \le Zscore_i^{LR_n} < 0 & 3 \\ 0 \le Zscore_i^{LR_n} < 1 & 4 \\ 1 \le Zscore_i^{LR_n} < 2 & 5 \\ 2 \le Zscore_i^{LR_n} < 3 & 6 \\ 3 \le Zscore_i^{LR_n} < 4 & 7 \\ 4 \le Zscore_i^{LR_n} < 5 & 8 \\ 5 \le Zscore_i^{LR_n} < 6 & 9 \\ \dots\dots & \dots\dots \\ n \le Zscore_i^{LR_n} < n+1\ (n \ge -3) & n+4 \end{array} \right\}$	(14)	Converting the $Zscore_i^{LR_n}$ into $ZT_i^{LR_n}$.
9.	$ZTTotal = \sum_{i=1}^{N} ZT_i^{LR_n}$	(15)	Calculating the total interaction intensity $ZTTotal$ for each ligand-receptor (LR_n) of cell

			subtypes and all the brain regions, respectively.
10.	$Density^{LR_n} = \frac{ZTTotalA/A}{ZTTotalB/B}$	(16)	Calculating the average $Density^{LR_n}$ for each ligand-receptor (LR_n) of cell subtypes.

Table 8 Cell-cell communication strength (Density) algorithm

**Algorithm** Cell-cell communication strength (Density) algorithm	
1:	**Input:** $Zscore_i^{LR_n}$
2:	**For** each $Zscore_i^{LR_n}$ of each ligand-receptor (LR_n) at each point i on the cell subtypes (A points) and all the brain regions section (B points), index *n*：
3:	Convert these two $Zscore_i^{LR_n}$ into ZT_A_i and ZT_B_i by Eq.14
4:	**For** each ZT_A_i and ZT_B_i of LR_n, index *i*：
5:	Calculate the total interaction intensity $ZTTotalA$ and $ZTTotalB$ by Eq.15
6:	**For** $ZTTotalA$ and $ZTTotalB$ of LR_n：
7:	Calculate the average $Density^{LR_n}$ for LR_n of cell subtypes by Eq.16
8:	**Output**: $Density^{LR_n}$

Table 9 Parameter Definition for Similarity algorithm for mutual pathway sets

No.	Definition		Description
1.	$P\_A_{Trajectory_n}, (n = 1 \dots N)$		Mutual pathway sets for our identified cell subtypes (SR6. Table 2.1-2.3 and SR6. Figure 1.1-1.3).
2.	$P\_B_{Trajectory_n}, (n = 1 \dots N)$		Mutual pathway sets for similar cell types (SR6. Table 3.1-3.3 and SR6. Figure 2.1-2.3).
3.	$P\_inter_{Trajectory_n} = P\_A_{Trajectory_n} \cap P\_B_{Trajectory_n}$	(17)	Mutual pathway set of our identified cell subtypes and similar cell types (SR6. Table 4).
4.	$Similarity_{Trajectory_n}^{P_{A\_B}} = \frac{P\_inter_{Trajectory_n}}{P\_A_{Trajectory_n}}$	(18)	The similarity between $P\_A_{Trajectory_n}$ and $P\_B_{Trajectory_n}$ (SR6. Figure 3).

Table 10 Similarity algorithm for mutual pathway sets

**Algorithm** Similarity algorithm for mutual pathway sets	
1:	**Input:** $P\_A_{Trajectory_n}, P\_B_{Trajectory_n}, (n = 1 \dots N)$
2:	**For** each $P\_A_{Trajectory_n}$, $P\_B_{Trajectory_n}$, index *n*：
3:	Calculate the mutual pathway set $P\_inter_{Trajectory_n}$ between $P\_A_{Trajectory_n}$ and $P\_B_{Trajectory_n}$ by Eq.17
4:	**For** each $P\_inter_{Trajectory_n}$, index *n*：
5:	Calculate the similarity $Similarity_{Trajectory_n}^{P_{A\_B}}$ by Eq.18
6:	**Output**: $P\_inter_{Trajectory_n}$, $Similarity_{Trajectory_n}^{P_{A\_B}}$
